# Comprehensive analysis of PD-L1 expression, tumor-infiltrating lymphocytes, and tumor microenvironment in LUAD: differences between Asians and Caucasians

**DOI:** 10.1186/s13148-021-01221-3

**Published:** 2021-12-21

**Authors:** Fenglong Bie, He Tian, Nan Sun, Ruochuan Zang, Moyan Zhang, Peng Song, Lei Liu, Yue Peng, Guangyu Bai, Bolun Zhou, Shugeng Gao

**Affiliations:** 1 Department of Thoracic Surgery, National Cancer Center/National Clinical Research Center for Cancer/Cancer Hospital, Chinese Academy of Medical Sciences and Peking Union Medical College, NO. 17, Panjiayuannanli, Chaoyang District, Beijing, 100021 China; 2 State Key Laboratory of Molecular Oncology, National Cancer Center/National Clinical Research Center for Cancer/Cancer Hospital, Chinese Academy of Medical Sciences and Peking Union Medical College, Beijing, 100021 China

**Keywords:** Lung adenocarcinoma (LUAD), PD-L1 expression, Tumor-infiltrating lymphocytes (TILs), Tumor microenvironment (TME), Asians and Caucasians

## Abstract

**Backgrounds:**

The characteristics of programmed cell death protein-1 (PD-L1) expression, tumor-infiltrating lymphocytes (TILs), and tumor microenvironment (TME) in lung adenocarcinoma (LUAD) patients are closely related to immunotherapy, and there are differences between Asians and Caucasians.

**Methods:**

Acquire the transcriptome data of the Cancer Genome Atlas and Chinese LUAD patients. R software was used to analyze the differential expression of genes, prognosis, and gene function. Use CIBERSORT for TIL-related analysis and ESTIMATE for TME-related analysis.

**Results:**

The expression of PD-L1 in tumor tissues of Caucasian LUAD patients was lower than that in normal tissues, while there was no significant difference in Asians. There was no statistical difference between PD-L1 expression and prognosis. The composition of TILs between Caucasian and Asian LUAD patients was quite different. There was no correlation between TILs and prognosis in Caucasians. However, the higher content of resting mast cells indicated a better prognosis in Asians. The Caucasian patients with higher immune and estimate scores had a better prognosis (*p* = 0.021, *p* = 0.025). However, the Asian patients with a higher estimate score had a worse prognosis (*p* = 0.024). The high expression of COL5A2 (*p* = 0.046, *p* = 0.027) and NOX4 (*p* = 0.020, *p* = 0.019) were both associated with the poor prognosis in Caucasians and Asians.

**Conclusion:**

There are many differences in the characteristics of PD-L1 expression, TILs, and TME between Caucasian and Asian LUAD patients. This provides a certain hint for the selection of specific immunotherapy strategies separately for Caucasian and Asian LUAD patients.

**Supplementary Information:**

The online version contains supplementary material available at 10.1186/s13148-021-01221-3.

## Introduction

Lung cancer is the cancer with the highest fatality rate and the second-highest incidence rate in the world [1, 2]. Lung cancer mainly includes non-small cell lung cancer (NSCLC) and small cell lung cancer (SCLC), and non-small cell lung cancer accounts for 85% [3]. The main pathological types of NSCLC are lung adenocarcinoma (LUAD), lung squamous cell carcinoma (LUSC), and large cell carcinoma. In recent years, the proportion of LUAD is increasing, and it has become the most common pathological type of NSCLC [4]. There are many treatment methods for lung cancer, such as radiotherapy, chemotherapy, targeted therapy, immunotherapy, and immunotherapy has achieved good therapeutic effects in lung cancer [5, 6]. At present, many studies on immune checkpoint inhibitors (ICIs) therapy are based on Western databases such as the Cancer Genome Atlas (TCGA), and there are few studies comparing the differences between Asians and Caucasians. This study took LUAD as the research disease, focusing on the analysis of immunotherapy-related programmed death-ligand 1 (PD-L1, also called CD274) expression, tumor-infiltrating lymphocytes (TILs), and tumor microenvironment (TME) characteristics between Asians and Caucasians.

Immunotherapy that emerged in recent years has achieved good results in the treatment of NSCLC [7, 8]. The situation of immunotherapy in LUAD is slightly more complicated due to the existence of various driver gene mutations [9]. The current studies have shown that the LUAD patients with mutations such as EGFR are less effective in receiving ICIs therapy, which may be related to the lower expression of PD-L1 and lower level of tumor mutational burden (TMB) [10]. At present, most reports indicate that the LUAD patients with mutations such as KRAS have a better effect on ICIs therapy, but the situation for different KRAS subtypes is also inconsistent [11]. Moreover, the situation is different in other tumors, and there are still many controversies [12]. This study took LUAD as the research disease and studied the characteristics of PD-L1 expression, TILs, and TME related to immunotherapy in LUAD.

There are many studies on anti-PD-L1/PD-1 ICIs therapy of lung cancer, which have grown rapidly in recent years [13, 14]. Most of the studies are based on Western clinical trial data, and in recent years, there have been more related clinical trials in the East. However, few studies are focusing on comparing the differences between the Eastern and Western. The Western countries are dominated by Caucasian whites, while the Eastern countries are dominated by yellow races. The genetic differences are obvious between Caucasians and Asians. For example, the most common LUAD mutation types in Western countries are TP53, KRAS, STK11, and EGFR [15], while in Asians is EGFR [16–18]. The characteristics of PD-L1 expression, TILs, and TME should be different between Caucasians and Asians due to the different genetic makeup. This study focused on comparing the differences between Caucasians and Asians on the characteristics of PD-L1 expression, TILs, and TME related to immunotherapy in LUAD.

The treatment methods of LUAD include surgery, radiotherapy, chemotherapy, targeted therapy, and immunotherapy, but each method has its limitations [19]. Immunotherapy refers to a treatment method that artificially enhances or suppresses the immune function of the body to achieve the purpose of curing diseases [20]. Tumor immunotherapy aims at activating the human immune system, relying on the autoimmune function to kill tumor cells, and has a huge advantage in the treatment of tumors [21]. However, there are still many problems regarding the application of immunotherapy in LUAD, and there are also many differences between the Eastern and Western [14]. This study took LUAD as the research disease and focused on comparing the differences between Caucasians and Asians in the characteristics of PD-L1 expression, TILs, and TME related to immunotherapy.

## Patients and methods

### Data download

Download the Caucasian LUAD transcriptome data (normal 59, tumor 535) and related clinical information from the TCGA database (https://portal.gdc.cancer.gov/), and use R software (R x64 4.1.0) for processing to get mRNA matrix. Download the Asian LUAD transcriptome data (normal 49, tumor 51) and related clinical information [22] from the article published by Xu JY et al., and use R software for similar processing.

### Differential gene expression and prognostic analysis

For the mRNA matrix data obtained above, R software was used for gene differential expression analysis, and to draw heatmaps, deviation plots, scatter plots, paired scatter plots, and barplots. Use R software to draw Venn plots to intersect genes, and to analyze prognosis by combining the transcriptome and clinical information. To analyze the impact of a single factor on survival, log-rank tests were performed, and Kaplan–Meier survival curves were drawn. To evaluate the impact of multiple factors on survival, R software was used to perform univariate and multivariate Cox proportional hazards regression analysis. Draw corresponding forest plots and radar plots for the results of COX analysis.

### Gene function enrichment analysis

Use Gene Set Enrichment Analysis (GSEA) software (4.1.0) to perform the gene function enrichment analysis on transcriptome data. The gene sets of GSEA used in this study include Gene Ontology (GO, c5.all.v7.4.symbols.gmt [Gene ontology]), Kyoto Encyclopedia of Genes and Genomes (KEGG, c2.cp.kegg.v7.4.symbols.gmt [Curated]), and Immunologic signatures (c7.all.v7.4.symbols.gmt [Immunologic signatures]). Use R software to draw GO and KEGG circle plots, bar plots, and bubble plots.

### TILs analysis

TILs refer to the infiltrating immune cells that can be isolated from tumor tissues. The deconvolution algorithm CIBERSORT was used to predict the TILs composition of complex tissues based on standardized gene expression data [23]. This study used R software to perform CIBERSORT calculation to estimate the type and content of TILs in tumors. R software was used to draw barplots, heatmaps, correlation heatmaps, correlation circle plots, vioplots, and other graphics.

### TME analysis

TME is the cellular environment in which tumor cells are located, and its composition includes extracellular matrix, soluble molecules, tumor stromal cells, and so on. Numerous immune cells will be chemotaxis into TME and participate in the formation of TME. In TME, the immune and stromal cells are two main types of non-tumor components, which are of great value for tumor diagnosis and prognosis. In this study, ESTIMATE (Estimation of stromal and immune cells in malignant tumor tissues using expression data) was used to analyze the gene expression data to predict the tumor purity (estimate score), stromal component content (stromal score), and immune cell content (immune score) in TME [24]. ESTIMATE is a method that uses gene expression characteristics to infer the ratio of interstitial and immune cells in tumor samples. The expression data can be used to estimate the content of stromal and immune cells in malignant tumor tissues through the ESTIMATE algorithm. Predict the immune score and stromal score, thereby predicting its content, and calculate the tumor purity of each tumor sample. If the content of stromal cells and immune cells is high, the purity of the tumor is low. On the contrary, the purity of the tumor is high.

## Results

### PD-L1 expression and clinical features

**Fig. 1:**
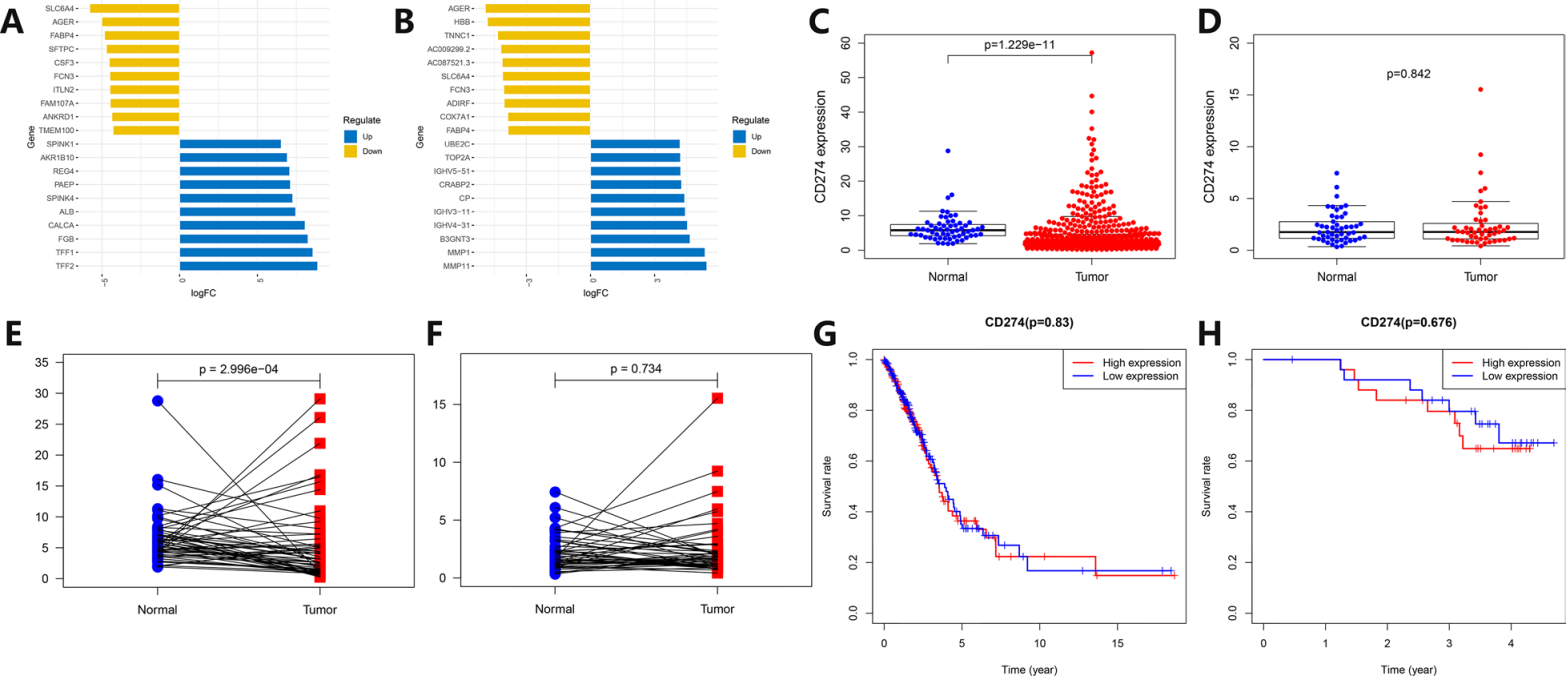
Analysis of differentially expressed genes and correlation between PD-L1 expression and prognosis in Caucasian and Asian LUAD patients. **A**, **B** Deviation plots of low and high expression differential genes in tumor and normal tissues in Caucasian (left) and Asian (right) LUAD patients. The abscissa represents logFC values, and the ordinate represents gene names. The dark blue represents tumor up-regulated genes, and the brown represents down-regulated genes in tumor tissues. **C**, **D** Scatter plots of PD-L1 expression in tumor and normal tissues in Caucasian (left) and Asian (right) LUAD patients. The abscissa represents the grouping of tumor and normal tissues, and the ordinate represents the relative PD-L1 expression. The blue represents normal tissues, and the red represents tumor tissues. **E**, **F** Paired scatter plots of PD-L1 expression in tumor and normal tissues in Caucasian (left) and Asian (right) LUAD patients. The abscissa represents the grouping of tumor and normal tissues, and the ordinate represents the PD-L1 relative expression. The blue represents normal tissues, and the red represents tumor tissues. **G**, **H** Survival curves of PD-L1 expression in tumor tissues in Caucasian (left) and Asian (right) LUAD patients. The abscissa represents survival time, and the ordinate represents survival rate. The red represents the PD-L1 high expression group, and the blue represents the PD-L1 low expression group

The transcriptome data of Caucasian and Asian LUAD were divided into two groups according to the tumor and normal tissues, and the differential expression analysis was performed to draw heatmaps (Additional file 1: Fig. S1A, B). The top 10 genes were screened out according to logFC values, and deviation plots were made (Fig. 1A, B). Although the differentially expressed genes obtained from the Caucasian and Asian data were quite different, the top 10 downregulated genes both included AGER, FABP4, and FCN3. Extract PD-L1 expression data separately to draw scatter plots (Fig. 1C, D) and paired scatter plots (Fig. 1E, F). The Caucasian data showed that the PD-L1 expression levels in normal tissues were higher than in tumor tissues. However, the data from Asians showed that the PD-L1 expression was not statistically different between tumor and normal tissues. The patients were divided into two groups according to the median expression of PD-L1, and survival curves were drawn. The survival curves of Caucasians and Asians (Fig. 1G, H) showed that there were no significant differences between PD-L1 expression and prognosis.

The patients were grouped according to different clinical factors, to explore the differences in different PD-L1 expression groups. The results are shown in Additional file 2: Fig. S2A–G. Barplots of clinical factors correlation analysis in Asians and Caucasians showed that the correlation between gender and PD-L1 expression of LUAD patients had statistical differences (*p* = 0.024, *p* = 0.022). However, the correlation between age (*p* = 0.343, *p* = 0.699), survival status (*p* = 0.445, *p* = 0.846), N stage (*p* = 0.343, *p* = 0.224), smoking status (*p* = 0.769, *p* = 0.055), clinical stage (*p* = 0.840, *p* = 0.556), T stage (*p* = 0.356, *p* = 0.213) and PD-L1 expression of LUAD patients had no statistical difference.

**Fig. 2:**
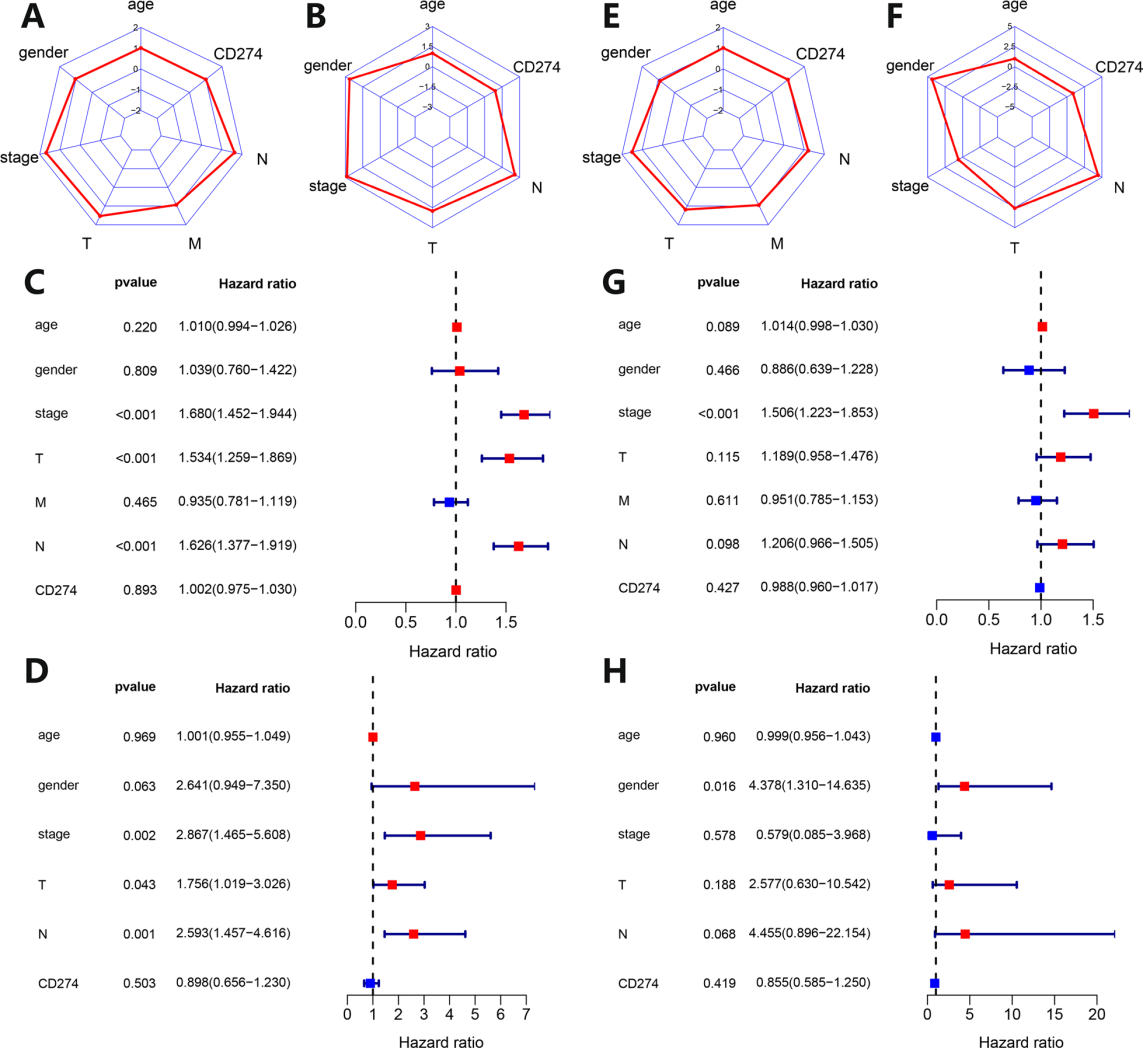
Univariate and multivariate COX proportional hazards regression analysis of PD-L1 expression and clinical characteristics in tumor tissues in Caucasian and Asian LUAD patients. **A**, **B** Radar plots of univariate COX analysis of PD-L1 expression and clinical characteristics in tumor tissues in Caucasian (left) and Asian (right) LUAD patients. The surrounding coordinates represent the PD-L1 expression in tumor tissue and clinical characteristics, and the coordinate axis scale represents the hazard ratio of univariate COX analysis. **C**, **D** Forest plots of univariate COX analysis of PD-L1 expression and clinical characteristics in tumor tissues in Caucasian (up) and Asian (down) LUAD patients. **E**, **F** Radar plots of PD-L1 expression and clinical characteristics in tumor tissues in Caucasian (left) and Asian (right) LUAD patients. The surrounding coordinates represent the PD-L1 expression in tumor tissue and clinical characteristics, and the coordinate axis scale represents the hazard ratio of multivariate COX analysis. **G**, **H** Forest plots of multivariate COX analysis of PD-L1 expression and clinical characteristics in tumor tissues in Caucasian (up) and Asian (down) LUAD patients

The PD-L1 expression and related clinical factors were subjected to univariate and multivariate Cox proportional hazards regression analysis, and the results were plotted into radar and forest plots. The radar plots (Fig. 2A, B) and forest plots (Fig. 2C, D) of univariate Cox proportional hazards regression analysis showed that whether for Caucasian or Asian LUAD, clinical stage, T stage, and N stage were all factors that affect prognosis. The radar plots (Fig. 2E, F) and forest plots (Fig. 2G, H) of multivariate Cox proportional hazards regression analysis (all variables of univariate analysis included in multivariate analysis) showed that only the clinical stage was a factor that independently affects prognosis in Caucasians. For Asians, only gender was an independent prognostic factor.

### GSEA analysis of PD-L1 expression

**Fig. 3:**
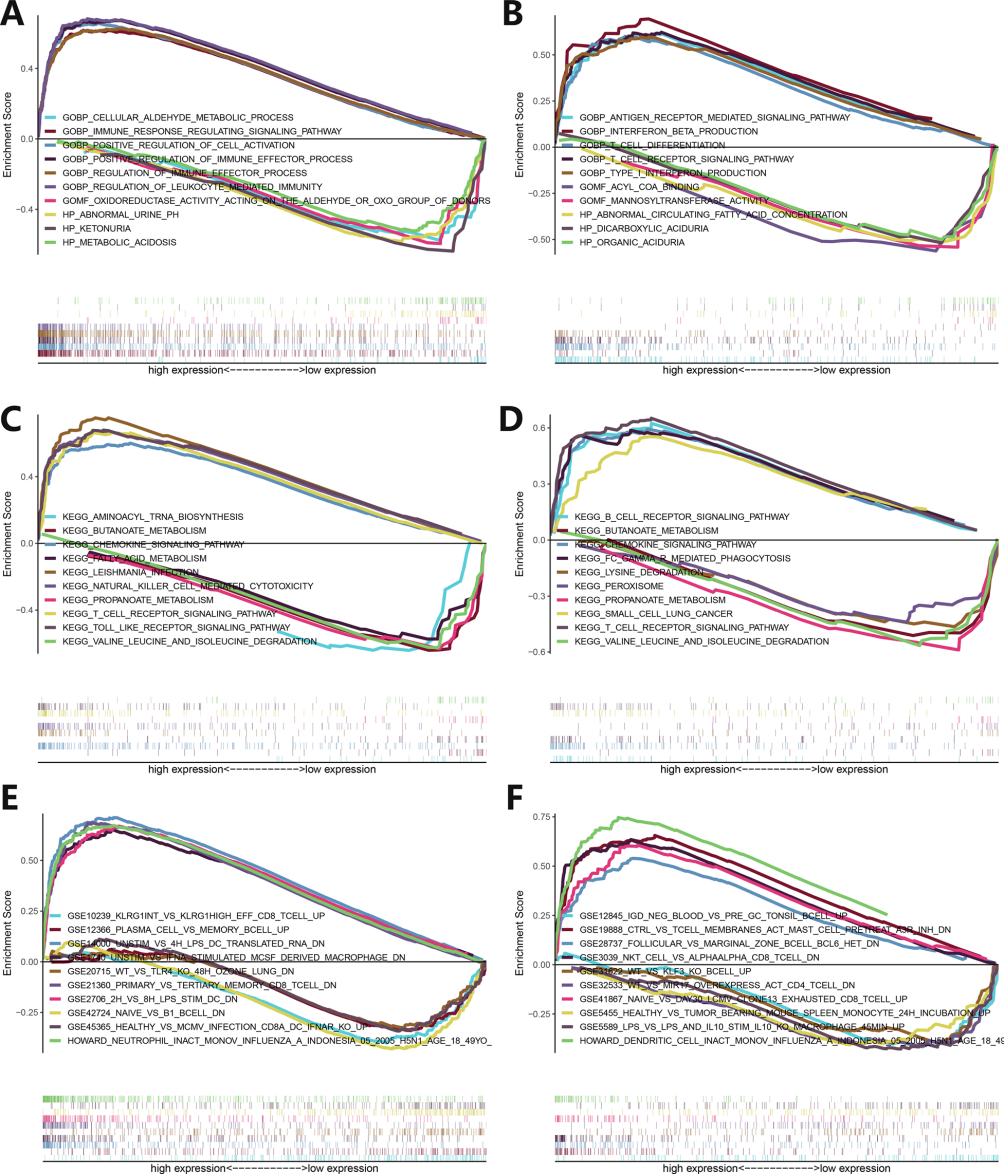
GSEA enrichment analysis of functional pathways related to PD-L1 expression in tumor tissues in Caucasian and Asian LUAD patients. The abscissa represents gene expression, and the ordinate represents enrichment score. **A**, **B** GSEA enrichment analysis of GO functional pathways related to PD-L1 expression in tumor tissues in Caucasian (left) and Asian (right) LUAD patients. **C**, **D** GSEA enrichment analysis of KEGG functional pathway related to PD-L1 expression in tumor tissues in Caucasian (left) and Asian (right) LUAD patients. **E**, **F** GSEA enrichment analysis of Immunologic Signature functional pathways related to PD-L1 expression in tumor tissues in Caucasian (left) and Asian (right) LUAD patients

The transcriptome data of LUAD patients were sorted according to the expression of PD-L1 and divided into the PD-L1 high and low expression groups. GSEA analysis was performed of GO, KEGG, and Immunologic Signatures sets. The GO analysis from Caucasian patients showed that the signal pathways enriched in the PHE and PLE groups were different, the former was mainly enriched in positive regulatory cell activation, immune effect, immune signal-related pathways, and the latter was enriched in urine, aldehyde, ketonuria metabolism-related pathways (Fig. 3A). The enriched signaling pathways of Asians in the PHE and PLE groups were different, the former was mainly enriched in interferon production, T cell differentiation, T cell receptor, antigen receptor-mediated signaling pathway, and the latter was enriched in aciduria, mannosyl, fatty acid metabolism (Fig. 3B). The KEGG analysis from Caucasian patients showed that the signal pathways enriched in PHE and PLE groups were different, the former was mainly enriched in toll-like receptor, NK cell-mediated cytotoxicity, T cell receptor, chemokine, other immune-related signaling pathways, and the latter was enriched in butanoate, propanoate, valine leucine, isoleucine, fatty acid metabolism pathway (Fig. 3C). The enriched signaling pathways of Asians in the PHE and PLE groups were different. The former was mainly enriched in T cell receptor, B cell receptor, phagocytosis, chemokine signaling pathway, and the latter was enriched in valine, leucine, isoleucine, propanoate, lysine, butanoate metabolism (Fig. 3D). The Immunologic Signatures analysis from Caucasian patients showed that the signal pathways enriched in PHE and PLE groups were different, the former was mainly enriched in monocytes, dendritic cells, neutrophil, memory CD8+ T cell-related experiments, and the latter was enriched in B lymphocytes, effector CD8+ T cells, dendritic cells related experiments (Fig. 3E). The enriched signaling pathways of Asians in the PHE and PLE groups were different, the former was mainly enriched in CD8+ T cells, NKT cells, B cells signaling pathway, and the latter was enriched in B lymphocytes, macrophages, CD4+ T cells, T lymphocytes signaling pathway (Fig. 3F).

### TILs analysis

**Fig. 4:**
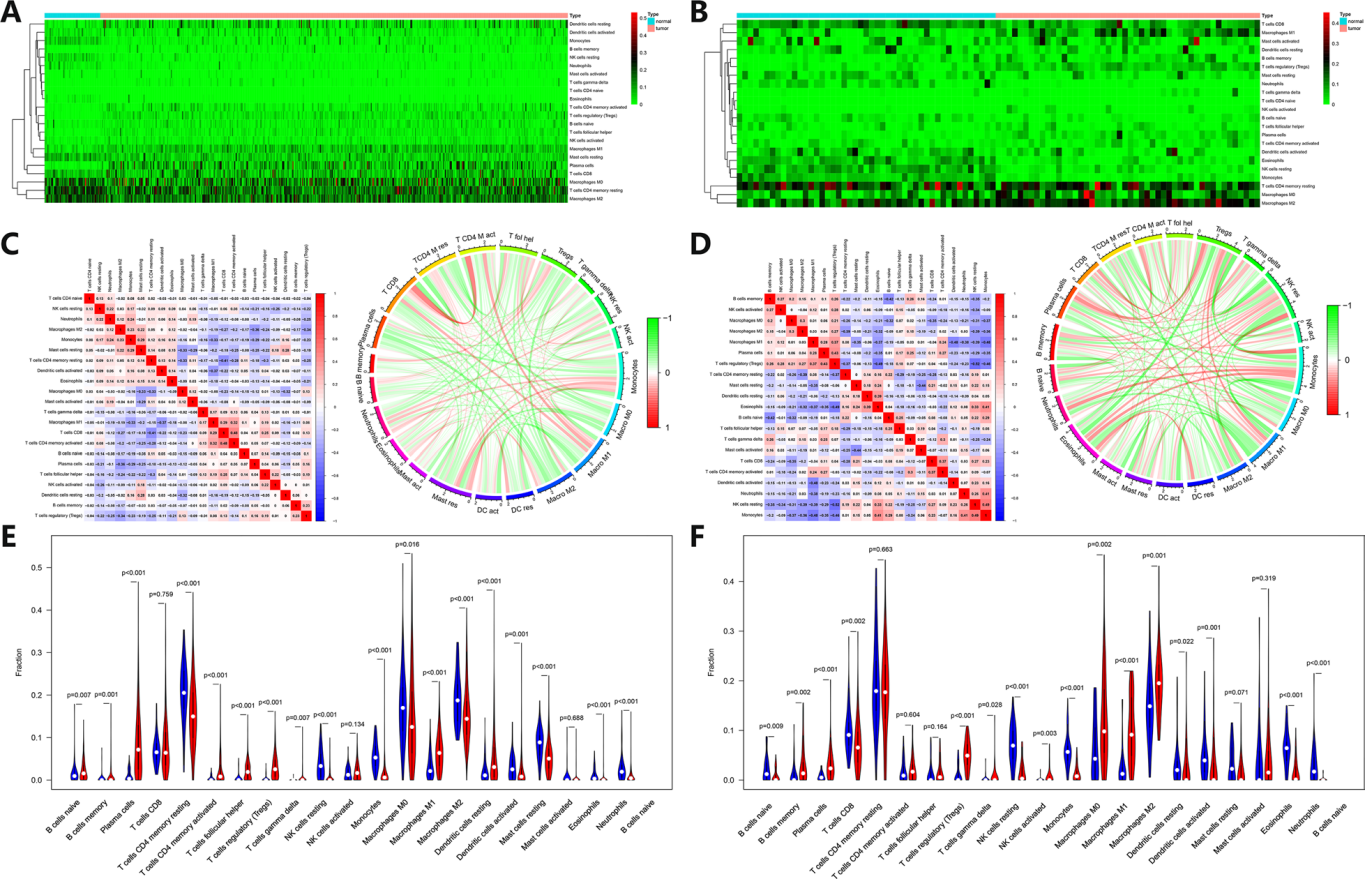
Analysis of TILs in Caucasian and Asian LUAD patients. **A**, **B** Heatmaps of the relative TILs content in Caucasian (left) and Asian (right) LUAD patients. The abscissa represents patients’ ID, and the ordinate represents the type of TILs. The blue bar in the first row represents normal tissues, and the red bar represents tumors. The green represents the relatively low content of TILs, and the red represents the relatively high content of TILs. **C** Correlation analysis of TILs in tumors of LUAD patients in Caucasians. Left: correlation heatmap of TILs. The abscissa and ordinate represent the names of TILs. The red represents positive correlation, and the blue represents negative correlation. Right: correlation circle plot of TILs. The outer coordinate represents the names of TILs. The red represents positive correlation, and the green represents negative correlation. **D** Correlation analysis of TILs in tumors of Asian LUAD patients. Left: correlation heatmap of TILs. The abscissa and ordinate represent the names of TILs. The red represents positive correlation, and the blue represents negative correlation. Right: correlation circle plot of TILs. Outer coordinate represents the names of TILs. The red represents positive correlation, and the green represents negative correlation. **E**, **F** Vioplots of relative TILs content in Caucasian (left) and Asian (right) LUAD patients. The abscissa represents the type of TILs, and the ordinate represents the percentage of relative TILs content. The blue column represents normal tissues, and the red column represents tumor tissues

The CIBERSORT algorithm was used to predict the composition of TILs in tumor tissue. The barplots (Additional file 3: Fig. S3A, B) and heatmaps (Fig. 4A, B) showed that the expression profiles of TILs in Caucasian and Asian LUAD tumor tissues were different. Plasma cells, T follicular helper cells, T regulatory cells (Tregs), M1 macrophages, and resting dendritic cells were the more abundant TILs types in Caucasian LUAD, while B memory cells, plasma cells, Tregs, M0 macrophages, M1 macrophages, and M2 macrophages were the more abundant TILs types in Asian LUAD. The Caucasian LUAD tumor tissues contained fewer resting CD4+ T memory cells, resting NK cells, monocytes, M0 macrophages, M2 macrophages, resting mast cells, and neutrophils, while the Asian LUAD tumor tissues contained fewer CD8+ T cells, resting NK cells, monocytes, activated dendritic cells and eosinophils.

Perform the correlation analysis on TILs expression profile data, and draw the correlation heatmaps and circle plots (Fig. 4C, D). The correlation analysis based on Caucasian LUAD patients showed the following TILs combinations were highly positively correlated in statistics: CD8+ T cells combined with activated CD4+ T memory cells (*r* = 0.48), M1 macrophages combined with activated CD4+ T memory cells (*r* = 0.32), monocytes combined with resting mast cells (*r* = 0.29), and M1 macrophages combined with CD8+ T cells (*r* = 0.29). The following TILs combinations were highly negatively correlated in statistics: resting CD4+ T memory cells combined with CD8+ T cells (*r* = − 0.41), activated dendritic cells combined with M1 macrophages (*r* = − 037), and M2 macrophages combined with plasma cells (*r* = − 0.36). The correlation analysis based on Asian LUAD patients showed the following TILs combinations were highly positively correlated in statistics: resting NK cells combined with monocytes (*r* = 0.49), plasma cells combined with Tregs (*r* = 0.43), eosinophils combined with monocytes (*r* = 0.41), and neutrophils combined with monocytes (*r* = 0.41). The following TILs combinations were highly negatively correlated in statistics: Tregs combined with resting NK cells (*r* = − 0.52), Tregs combined with eosinophils (*r* = − 0.49), M1 macrophages combined with activated dendritic cells (*r* = − 0.48), and M1 macrophages combined with monocytes (*r* = − 0.48).

The vioplots (Fig. 4E, F) showed that TILs with higher content in Caucasian LUAD were naive B cells (*p* = 0.007), memory B cells (*p* = 0.001), plasma cells (*p* < 0.001), activated memory CD4+ T cells (*p* < 0.001), T follicular helper cells (*p* < 0.001), Tregs (*p* < 0.001), gamma delta T cells (*p* = 0.007), M1 macrophages (*p* < 0.001), resting dendritic cells (*p* < 0.001), and TILs with lower content were naïve CD4+ T cells (*p* = 0.004), resting CD4+ memory T cells (*p* < 0.001), resting NK cells (*p* < 0.001), monocytes (*p* < 0.001), M0 macrophages (*p* = 0.016), M2 macrophages (*p* < 0.001), activated dendritic cells (*p* = 0.001), resting mast cells (*p* < 0.001), eosinophils (*p* < 0.001) and neutrophils (*p* < 0.001). The more abundant TILs in Asian LUAD are B memory cells (*p* = 0.002), plasma cells (*p* < 0.001), Tregs (*p* < 0.001), T gamma delta cells (*p* = 0.028), M0 macrophages (*p* = 0.002), M1 Macrophages (*p* < 0.001), and M2 macrophages (*p* = 0.001), while the less content TILs were B naïve cells (*p* = 0.009), CD8+ T cells (*p* = 0.002), resting NK cells (*p* < 0.001), monocytes (*p* < 0.001), resting dendritic cells (*p* = 0.022), activated dendritic cells (*p* = 0.001), eosinophils (*p* < 0.001), and neutrophils (*p* < 0.001).

**Fig. 5:**
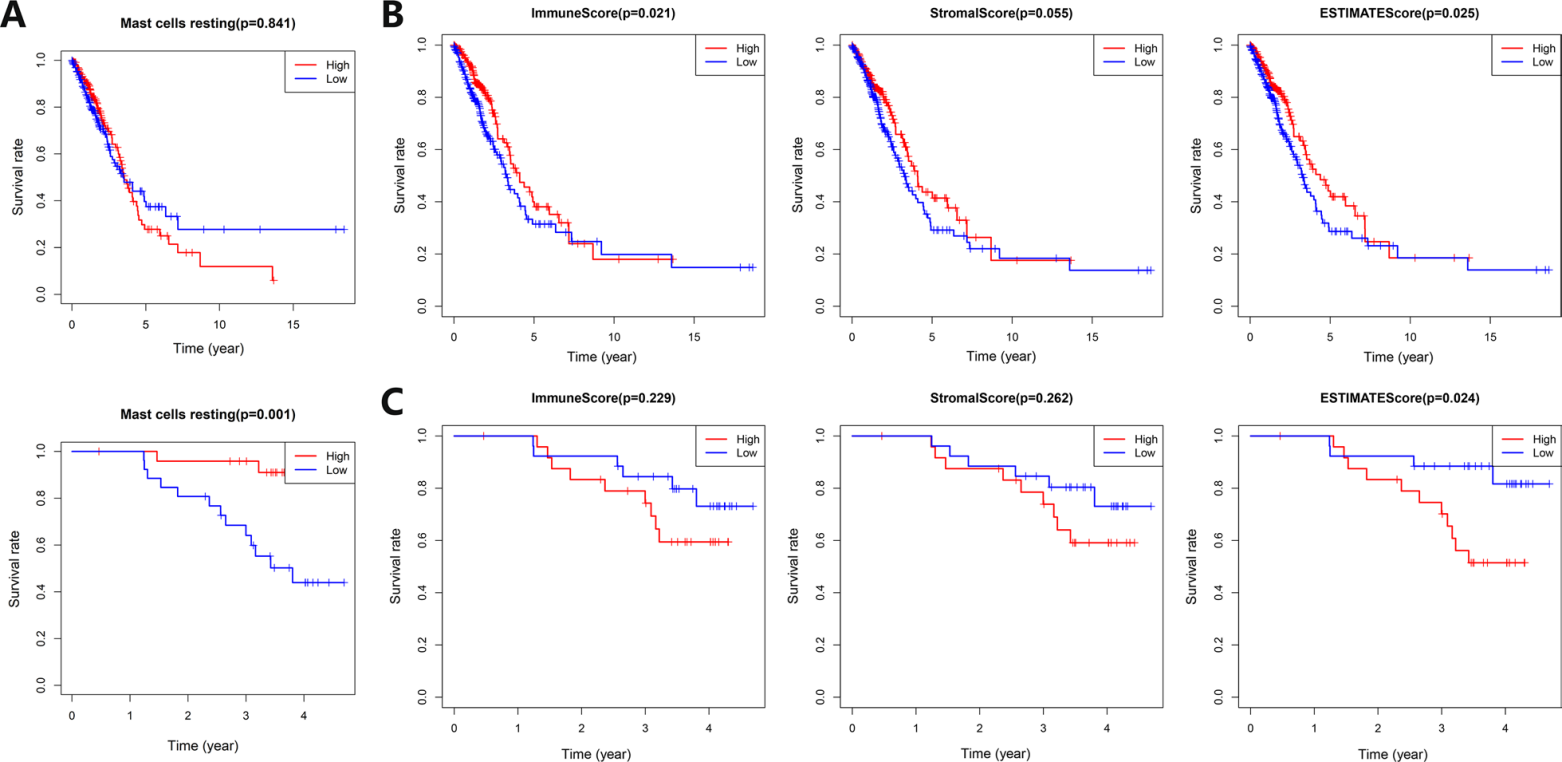
Prognostic analysis of TILs and TME-related scores in Caucasian and Asian LUAD patients. **A** Prognostic analysis of resting mast cells in Caucasian (up) and Asian (down) LUAD patients. The abscissa represents time, and the ordinate represents the survival rate. The red curve represents the group with more resting mast cells, and the blue curve represents the group with fewer resting mast cells. **B** Prognostic analysis of TME-related immune score (left), stromal score (middle), and estimate score (right) of Caucasian LUAD patients. The abscissa represents survival time, and the ordinate represents survival rate. The red line represents the high-scoring group, and the blue line represents the low-scoring group. **C** Prognostic analysis of TME-related immune score (left), stromal score (middle), and estimate score (right) of Asian LUAD patients. The abscissa represents survival time, and the ordinate represents survival rate. The red line represents the high-scoring group, and the blue line represents the low-scoring group

The patients were divided into groups according to the relative number of various TILs, to draw KM survival curves. The high content of resting mast cells in Asian LUAD patients indicated a better prognosis (Fig. 5A). The patients were grouped according to their clinical characteristics to explore the differences in the content of various TILs between different groups (Additional file 4: Fig. S4A–O). The age > 65-year group contained a higher M1 macrophages fraction (*p* = 0.026) in Caucasian LUAD patients, while the age > 65-year group contained a higher activated CD4+ T memory cells fraction (*p* = 0.031) in Asians. The alive group of Asian LUAD patients had a higher resting mast cells fraction (*p* = 0.012). The male group of Caucasian LUAD patients had a higher plasma cell fraction (*p* = 0.048). There were statistical differences in B memory cell fraction (*p* = 0.003), neutrophils fraction (*p* = 0.013), and activated dendritic cells fraction (*p* = 0.042) in different N stage groups of Caucasian LUAD patients. The no-smoking group of Caucasian LUAD patients contained higher resting dendritic cells fraction (*p* = 0.003), monocytes fraction (*p* = 0.007), and resting mast cells fraction (*p* = 0.012). The smoking group of Caucasian LUAD patients contained higher CD8+ T cells fraction (*p* < 0.001), activated CD4+ T memory cells fraction (*p* = 0.003), and M1 macrophages fraction (*p* = 0.004). B memory cell fraction (*p* = 0.007) of Caucasian LUAD patients in different clinical stage groups was statistically different. There were statistical differences in M2 macrophages fraction (*p* = 0.002) in different T stage groups of Caucasian LUAD patients.

### TME analysis

This study used the ESTIMATE tool to predict the tumor purity (estimate score), stromal component content (stromal score), and immune cell content (immune score) in TME. The patients were grouped according to TME-related scores, and KM survival curves were drawn (Fig. 5B, C). The Caucasian patients with higher immune and estimate scores had a better prognosis (*p* = 0.021, *p* = 0.025). However, the Asian patients with a higher estimate score had a worse prognosis (*p* = 0.024). For Caucasian and Asian LUAD patients, group according to age, survival status, gender, N stage, smoking status, clinical stage, T stage, and other clinical characteristics, and analyze differences of immune score, stromal score, and estimate score between different groups (Additional file 5: Fig. S5A–N).

### TME-related functional pathway analysis

**Fig. 6:**
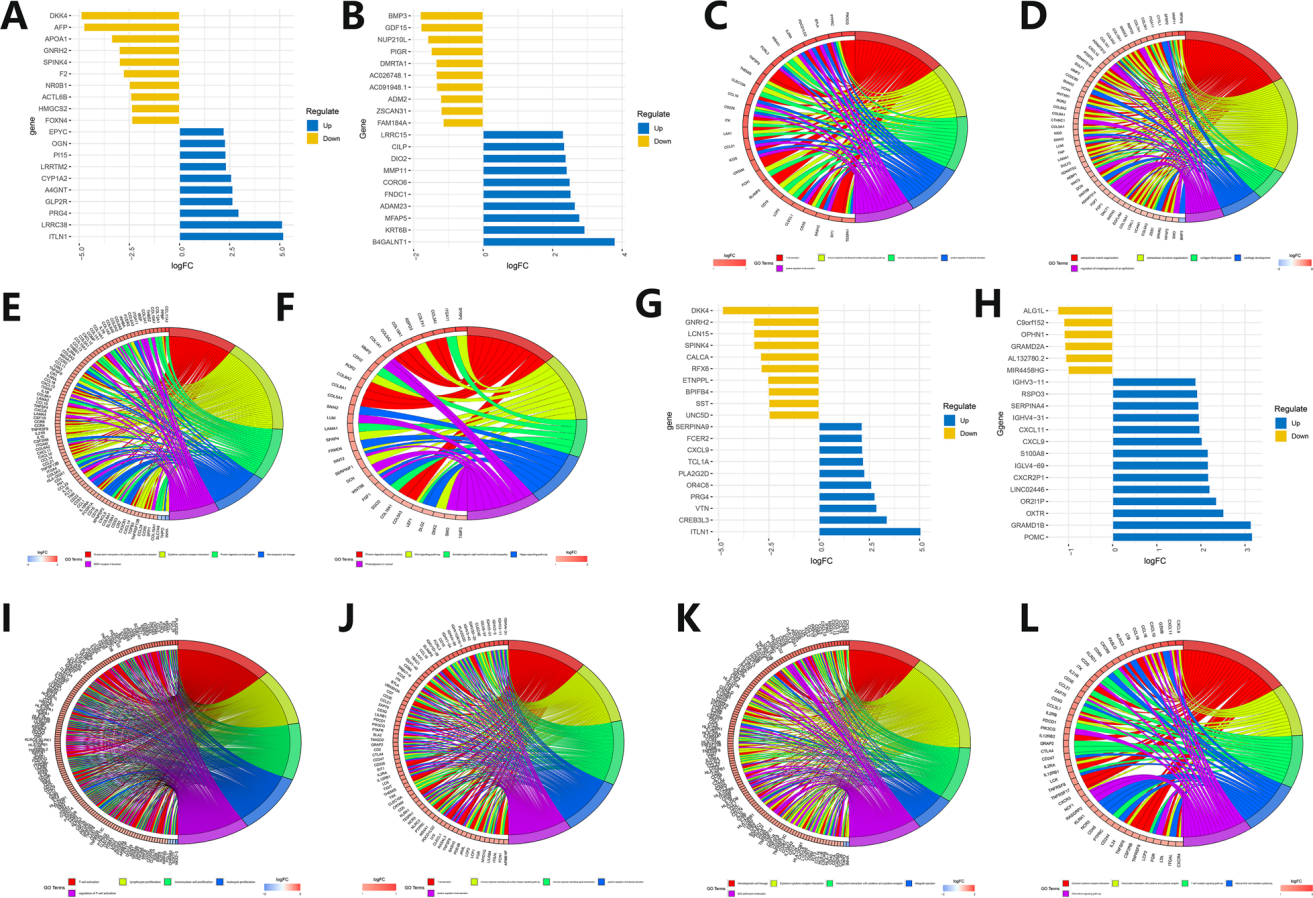
Differentially expressed genes and functional pathways enrichment analysis related to TME stromal and immune scores in Caucasian and Asian LUAD patients. **A**, **B** Deviation plot of differentially expressed genes related to TME stromal scores in Caucasian (left) and Asian (right) LUAD patients. The abscissa represents logFC values, and the ordinate represents gene names. The dark blue represents up-regulated genes in tumor tissues, and the brown represents down-regulated genes. A deviation plot is drawn based on the top 20 genes with the largest differences screened by logFC values. The abscissa represents logFC values, and the ordinate represents gene names. The dark blue represents up-regulated genes in the group with a higher TME stromal score, and the brown represents down-regulated genes. **C**, **D** GO circle plots of differentially expressed genes related to TME stromal scores in Caucasian (left) and Asian (right) LUAD patients. Five colors on the right side of the circle represent the 5 most enriched pathways, and the left coordinate represents enriched gene names and related logFC values. **E**, **F** KEGG circle plots of differentially expressed genes related to TME stromal scores in Caucasian (left) and Asian (right) LUAD patients. Five colors on the right side of the circle represent the 5 most enriched pathways, and the left coordinate represents enriched gene names and related logFC values. **G**, **H** Deviation plots of differentially expressed genes related to TME immune scores in Caucasian (left) and Asian (right) LUAD patients. The abscissa represents logFC values, and the ordinate represents gene names. The dark blue represents up-regulated genes in tumor tissues, and the brown represents down-regulated genes. Deviation plots were drawn based on the top 20 genes with the largest differences screened by logFC value. The abscissa represents logFC values, and the ordinate represents gene names. The dark blue represents up-regulated genes in the group with a higher TME immune score, and the brown represents down-regulated genes. **I**, **J** GO circle plots of differentially expressed genes related to TME immune scores in Caucasian (left) and Asian (right) LUAD patients. Five colors on the right side of the circle represent the 5 most enriched pathways, and the left coordinate represents enriched gene names and related logFC values. **K**, **L** KEGG circle plots of differentially expressed genes related to TME immune scores in Caucasian (left) and Asian (right) LUAD patients. Five colors on the right side of the circle represent the 5 most enriched pathways, and the left coordinate represents enriched gene names and related logFC values

The LUAD patients were divided into two groups according to the stromal score for gene differential expression analysis. Draw heatmaps for differentially expressed genes (Additional file 6: Fig. S6A, B), and screen out the top 20 genes with more obvious differences (10 low-expressed and 10 high-expressed genes) to draw deviation plots (Fig. 6A, B). The expression profiles of differential genes between Caucasian and Asian LUAD patients were quite different, and the top 20 genes with more obvious differences had no intersection. R software was used to make GO and KEGG circle plots of differentially expressed genes (Fig. 6C–F). The GO circle plots of Caucasian and Asian LUAD patients showed that they were mainly related to immune cells activating and extracellular matrix, respectively. The KEGG circle plots indicated that the Caucasian LUAD patients were mainly related to cytokine–cytokine receptor interaction, while the Asian LUAD patients were mainly related to protein digestion and absorption, and Wnt/Hippo signaling pathway.

Similarly, draw related heatmaps (Additional file 6: Fig. S6C, D), deviation plots (Fig. 6G, H), GO and KEGG circle plots (Fig. 6I–L) according to the immune score. The results of the GO circle plots in Caucasian and Asian LUAD patients showed that they were mainly related to immune cells activating and proliferation, and immune cells activating, respectively. The results of the KEGG circle plot showed that they were both mainly related to cytokine–cytokine receptor interaction.

**Fig. 7:**
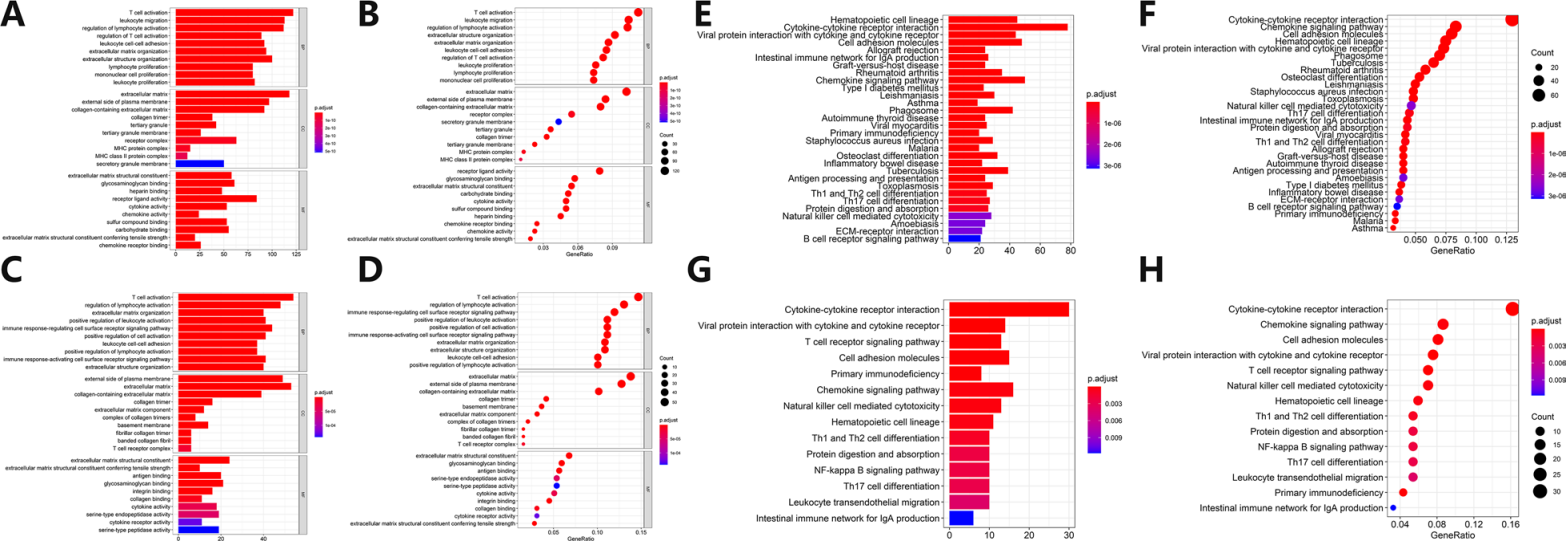
Enrichment analysis of functional pathways of differentially expressed genes related to TME stromal and immune scores in Caucasian and Asian LUAD patients. **A**–**D** GO barplots (left) and bubble plots (right) of functional pathways enrichment analysis of TME stromal and immune scores-related differentially expressed genes in Caucasian (up) and Asian (down) LUAD patients. Left: GO barplots for GO functional pathway enrichment analysis of differentially expressed genes related to TME scores. The abscissa represents gene count enriched in this pathway, and the ordinate represents the names of various pathways. The red represents a high degree of enrichment, and the blue represents a low degree of enrichment. Right: GO bubble plots for GO functional pathway enrichment analysis of differentially expressed genes related to TME scores. The abscissa represents gene ratio enriched to this pathway, and the ordinate represents names of various pathways. The red represents a high degree of enrichment, and the blue represents a low degree of enrichment. A larger circle represents a large number of genes enriched in this pathway, and a smaller circle represents a small number of genes enriched in this pathway. **E**–**H** KEGG barplots (left) and bubble plots (right) of functional pathways enrichment analysis of TME stromal and immune scores-related differentially expressed genes in Caucasian (up) and Asian (down) LUAD patients. Left: KEGG barplots for KEGG functional pathway enrichment analysis of differentially expressed genes related to TME scores. The abscissa represents gene count enriched in this pathway, and the ordinate represents the names of various pathways. The red represents a high degree of enrichment, and the blue represents a low degree of enrichment. Right: KEGG bubble plots for KEGG functional pathway enrichment analysis of differentially expressed genes related to TME scores. The abscissa represents gene ratio enriched to this pathway, and the ordinate represents names of various pathways. The red represents a high degree of enrichment, and the blue represents a low degree of enrichment. A larger circle represents a large number of genes enriched in this pathway, and a smaller circle represents a small number of genes enriched in this pathway

Take the intersection of the differential expression genes related to stromal and immune score, and then use R software to draw GO barplots, GO bubble plots, KEGG barplots, and KEGG bubble plots (Fig. 7A–H). For the Caucasian LUAD patients, the GO-related pathways mainly included immune cell activation and proliferation, extracellular matrix, and MHC complex. The KEGG-related pathways mainly included cytokine receptor interaction, immune-related diseases, and immune cell differentiation. For the Asian LUAD patients, the GO-related pathways mainly included immune cell activation, extracellular matrix, and cytokine activity. The KEGG-related pathways mainly included cytokine receptor interaction and immune cell differentiation.

### TME-related prognostic analysis

**Fig. 8:**
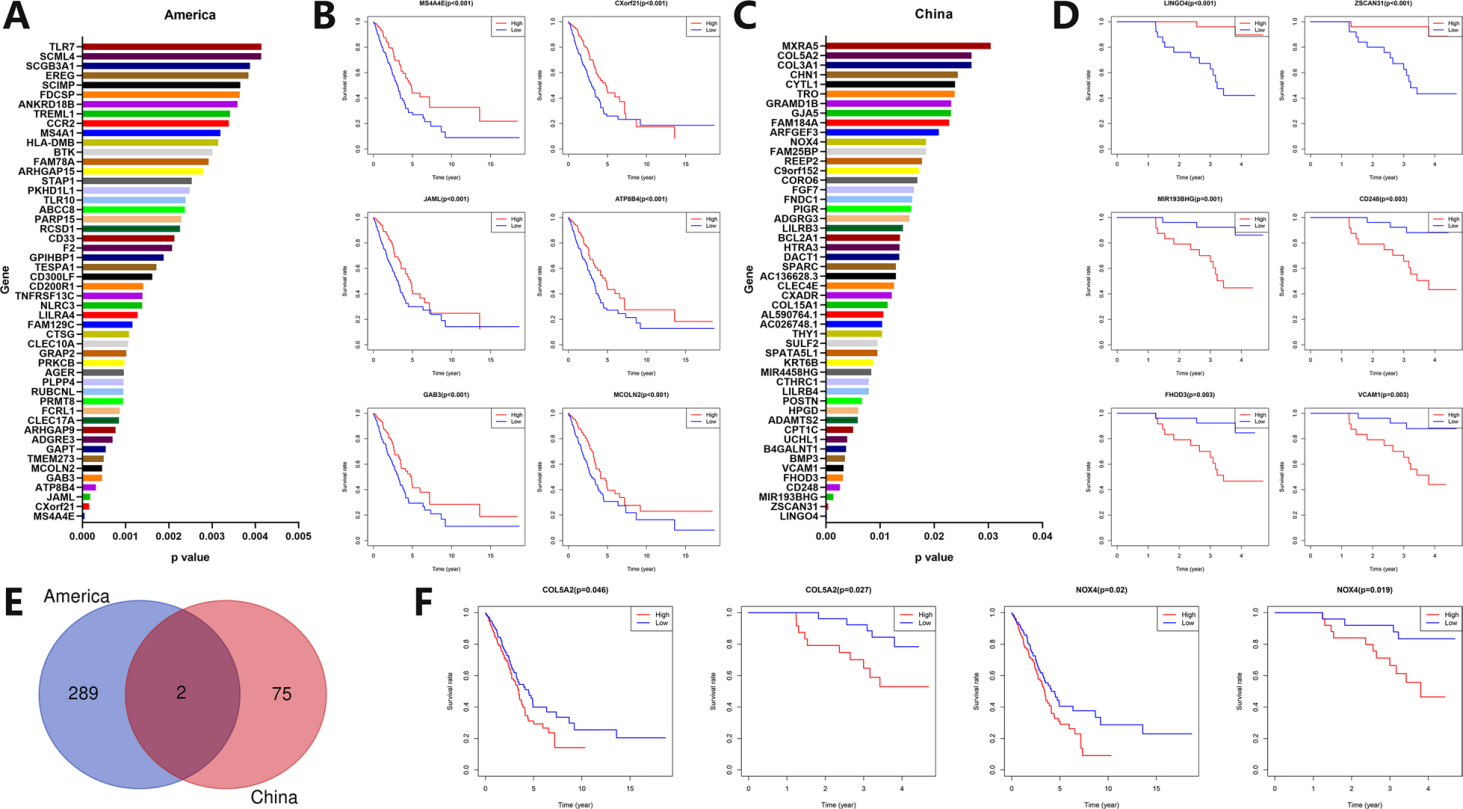
Analysis of TME stromal and immune scores related to differentially expressed genes and prognosis in Caucasian and Asian LUAD patients. **A** TME stromal and immune scores related to differentially expressed genes in Caucasian LUAD patients. The abscissa represents *p* values, and the ordinate represents gene names. Barplot shows 50 genes with smaller *p* values. **B** TME stromal and immune scores related prognostic survival curve of differentially expressed genes in Caucasian LUAD patients. The abscissa represents survival time, and the ordinate represents survival rate. The red line represents the high expression group, and the blue line represents the low expression group. **C** TME stromal and immune scores related to differentially expressed genes in Asian LUAD patients. The abscissa represents *p* values, and the ordinate represents gene names. Barplot shows 50 genes with smaller *p* values. **D** TME stromal and immune scores related prognostic survival curve of differentially expressed genes in Asian LUAD patients. The abscissa represents survival time, and the ordinate represents survival rate. The red line represents the high expression group, and the blue line represents the low expression group. **E** Venn plot of TME stromal and immune scores related differentially expressed genes in Caucasian and Asian LUAD patients. **F** Survival curves of TME stromal and immune scores related differentially expressed genes (COL5A2, NOX4) in Caucasian (left) and Asian (right) LUAD patients. The abscissa represents survival time, and the ordinate represents survival rate. The red line represents the high expression group, and the blue line represents the low expression group

The differentially expressed genes (FDR < 0.05, |logFC|> 1) related to the immune and stromal scores of Caucasian LUAD patients were screened out. According to FDR values, a histogram of the top 50 genes was drawn (Fig. 8A), showing survival curves of the top genes according to *p* values (Fig. 8B). A similar analysis was performed for Asian LUAD patients. Histogram (Fig. 8C) and survival curves (Fig. 8D) were drawn. The genes related to immune and stromal scores in Caucasian and Asian LUAD patients were screened out, and genes whose expression level was related to prognosis were drawn by Venn plot to obtain the intersection genes (Fig. 8E). Obtain two significantly prognostic genes, COL5A2 and NOX4, and display survival curves of the intersection genes (Fig. 8F). The high expressions of COL5A2 (*p* = 0.046, *p* = 0.027) and NOX4 (*p* = 0.020, *p* = 0.019) in Caucasian and Asian LUAD patients were both associated with poor prognosis.

## Discussion

Immunotherapy has become one of the main treatment methods of NSCLC [25, 26]. In recent years, the proportion of LUAD in lung cancer has gradually increased, and immunotherapy plays an important role in the treatment of LUAD [4, 27]. However, compared with LUSC, LUAD has a high proportion of EGFR, KRAS, and other driver gene mutations, with multiple mutation and co-mutations subtypes, and the selection of immunotherapy is more complicated [27]. There are great differences in LUAD between Asians and Caucasians. One of the most important aspects is the inconsistency of main driver genes. Caucasian LUAD is mainly driven by TP53, KRAS, LKB1, and other driver genes [15], while Asian LUAD is mainly driven by EGFR and other driver genes [16, 18]. Current studies have shown that the patients with EGFR, STK11, ALK, and other gene mutations are less effective in receiving ICIs therapy, while the patients with KRAS mutations have better therapeutic effects when receiving ICIs therapy, but the response of different mutation subtypes to immunotherapy is not consistent [28, 29]. Because of these factors, there are many differences in the predictive markers of immunotherapy efficacy, the choice of immunotherapy options, and the efficacy of immunotherapy between Asians and Caucasians. The efficacy of immunotherapy is closely related to PD-L1 expression, TILs, and TME. This study focuses on the differences between these factors in the East and West and hopes to bring certain hints to the immunotherapy of LUAD.

The tumor proportion score (TPS) method for PD-L1 expression evaluation has been included in NCCN guidelines (https://www.nccn.org/) as one of the criteria for screening NSCLC patients suitable for immunotherapy [30]. However, the conclusions of various studies on the efficacy prediction role of PD-L1 expression in immunotherapy are not completely consistent. The results of most current clinical trials show that the patients with high PD-L1 expression in tumors before therapy will have better immunotherapy effects. However, there are some patients with high PD-L1 expression, but the efficacy of immunotherapy is not ideal [31, 32]. On the contrary, some patients whose PD-L1 expression is low or even negative have better immunotherapy effects. The mechanisms of PD-L1 expression and immunotherapy efficacy are currently not very clear [33]. Anti-PD-1/PD-L1 ICIs can block the combination of PD-1/PD-L1 and exert its effect [34]. There is still controversy about the relationship between PD-L1 expression and prognosis, but most studies have shown that the high PD-L1 protein expression is associated with poor prognosis [35–38]. In this study, the relationship between PD-L1 expression and prognosis was analyzed from the mRNA level. The PD-L1 mRNA expression in tumor tissues of Caucasian LUAD patients was lower than that in normal tissues, while there was no statistically significant difference in Asian LUAD patients. There was no statistical difference between PD-L1 mRNA expression and prognosis analysis.

TILs play an important role in anti-tumor immunity and immunotherapy [39]. More and more results indicate that TILs in TME, such as T cells, NK cells, neutrophils, B cells, are closely related to the efficacy of receiving ICIs therapy [39]. Some studies have shown that patients with a large number of tumor-infiltrating CD8+ T cells before treatment have a better efficacy treated with ICIs [40]. Carcinoma-associated fibroblasts (CAFs), M2 macrophages, and Tregs in TME can inhibit the killing effect of CD8+ T cells on tumors and lead to depletion of CD8+ T cells. The use of ICIs can save depleted CD8+ T cells and restore its killing effect on tumor cells [41]. Results of this study showed that there was no statistical difference in the content of CD8+ T cells between tumor and normal tissues in Caucasian LUAD, while the content of CD8+ T cells in Asian LUAD tumors was less than that in normal tissues. The analyzing results of the CD8+ T cells content and prognosis were not statistically different both in Caucasian and Asian LUAD. For this result, we have conducted an analysis and believe that there are mainly the following reasons. Most of the current research results show that the more content of CD8+ T cells in the tumor, the better the treatment effect of ICIs, which has been confirmed [40, 42, 43]. However, the research on CD8+ T cells is focused on immunotherapy, and there are very few reports on the direct relationship between the content of CD8+ T cells and the prognosis. Therefore, the results of this study show that the content of CD8+ Tcells is not significantly correlated with the prognosis, which is reasonable. The content of CD8+ T cells in this study is calculated from transcriptome data using the CIBERSORT algorithm. Although the accuracy of this algorithm is relatively good, the content of CD8+ T cells is not acquired directly through immunohistochemistry, flow screening, immunofluorescence, or other testing methods. So, there will be a certain error. The sample size of this study is relatively small, which will also affect the results to a certain extent.

Immunohistochemistry (IHC), multiple immunofluorescences, and single-cell sequencing methods are usually used to observe the content of TILs in tumor tissues, and analyze the correlation between TILs and tumor occurrence, development, and prognosis [44]. With the development of machine learning, there are more intelligent means to analyze TILs in tumor tissues [45]. This study used the CIBERSORT deconvolution algorithm to calculate the content of TILs in tumor tissues by analyzing mRNA transcription data [23]. The composition of TILs between Caucasian and Asian LUAD patients was quite different. There was no correlation between the content of TILs and prognosis in Caucasians. However, the higher content of resting mast cells in Asian LUAD patients indicated a better prognosis.

TME is the microenvironment on which tumor cells grow and immune cells exert their anti-tumor effects [46]. Immune cells in TME play a very important role in the occurrence and development of tumors. They can usually inhibit tumor growth in the early stages of tumors, but immune escape will occur as the tumor progresses, which is one of the major characteristics of tumors [47]. The roles of stromal components and immune cells in TME are very complex. The heterogeneity of TME and immunotherapy in different tumors is very large, and even for the same tumor with a similar prognosis, the heterogeneity is also very large [48]. With the rise of immunotherapy, the relationship between TME and immunotherapy has taken on a new stage, and many studies struggle to improve the efficacy of immunotherapy by reshaping TME [49]. At present, many studies divide TME into four classic types by combining PD-L1 expression and TILs: Type I (adaptive immune resistance, PD-L1 (+), TIL (+)), Type II (immune ignorance, PD-L1 (−), TIL (−)), Type III (intrinsic induction, PD-L1 (+), TIL (−)), Type IV (tolerance, PD-L1 (−), TIL (+)) [50]. According to the ESTIMATE algorithm, this study scored stromal components and immune cells in TME by analyzing the mRNA transcriptome data and finally obtained the estimate score for evaluating the purity of the tumor [24]. This study showed that the Caucasian patients with higher immune and estimate scores had a better prognosis. However, the Asian patients with a higher estimate score had a worse prognosis (*p* = 0.024).

This study has many advantages. Focus on comparison in immunotherapy-related indicators between the East and West, which has a suggestive effect on the specific selection of ICIs treatment options between the East and West. Analyze differences in immunotherapy-related characteristics such as PD-L1 expression, TILs, and TME, and the analysis is systematic and comprehensive. This study simply analyzed transcriptome data at the mRNA level and carried out an extended analysis, which provides a better idea for the analysis of transcriptome data. There are also some shortcomings. The sample size is not very large and cannot fully represent differences between Asians and Caucasians. This article only analyzed the transcriptome data and lacks verification of proteome, IHC, and immunofluorescence. The PD-L1 protein level is more important than the mRNA level in the study of cancer immunotherapy. In the follow-up PD-L1-related research, we will pay more attention to the expression of the PD-L1 protein levels.

This study focused on PD-L1 expression, TILs, and TME and analyzed the differences in these characteristics between Asian and Caucasian LUAD patients. There are many differences between Asian and Caucasian LUAD patients in the expression of PD-L1 mRNA, the composition of TILs, the characteristics of TME, and the relationship between related clinical indicators and prognosis. The results provide certain hints for the selection of specific immunotherapy regimens separately for LUAD patients in the East and West. This research is based on the study of mRNA transcriptome data, and more reliable conclusions require further research.

## Supplementary Information

**Additional file 1: Fig. 1** Analysis of differentially expressed genes and correlation between PD-L1 expression and prognosis in Caucasian and Asian LUAD patients. (**A-B**) Differentially expressed gene heatmaps in tumor and normal tissues of LUAD patients in Caucasians (up) and Asians (down). The abscissa represents patients’ ID, and the ordinate represents gene names. The blue bar in the first row represents normal tissue, and the red bar represents tumor tissue. The green represents low-expressed genes, and the red represents high-expressed genes.

**Additional file 2: Fig. 2** Correlation analysis of PD-L1 expression and clinical characteristics in tumor tissues in Caucasian and Asian LUAD patients. (**A-G**) Barplots of correlation analysis in Caucasian (left) and Asian (right) LUAD patients between PD-L1 expression in tumor tissues and clinical characteristics such as age, survival status, gender, N stage, smoking status, clinical stage, and T stage. The abscissa represents clinical characteristics, and the ordinate represents PD-L1 relative expression.

**Additional file 3: Fig. 3** Analysis of TILs in Caucasian and Asian LUAD patients. (**A-B**) Barplots of TILs proportion in Caucasian (up) and Asian (down) LUAD patients. The abscissa represents patients’ ID, and the ordinate represents the percentage of each type of TILs.

**Additional file 4: Fig. 4** Clinical characteristics analysis of TILs in Caucasian and Asian LUAD patients. (**A-B**) Barplots of correlation analysis between M1 macrophages, activated CD4+ T memory cells, and age in Caucasian (left) and Asian (right) LUAD patients. (**C**) Barplots of correlation analysis between resting mast cells and survival status in Caucasian (left) and Asian (right) LUAD patients. (**D**) Barplots of correlation analysis between plasma cells and gender in Caucasian (left) and Asian (right) LUAD patients. (**E–G**) Barplots of correlation analysis between B memory cells, neutrophils, activated dendritic cells, and N stage in Caucasian (left) and Asian (right) LUAD patients. (**H-M**) Barplots of correlation analysis between CD8+ T cells, resting dendritic cells, activated CD4+ T memory cells, M1 macrophages, monocytes, resting mast cells, and smoking status in Caucasian (left) and Asian (right) LUAD patients. (**N**) Barplots of correlation analysis between B memory cells and clinical stage in Caucasian (left) and Asian (right) LUAD patients. (**O**) Barplots of correlation analysis between M2 macrophages and T stage in Caucasian (left) and Asian (right) LUAD patients.

**Additional file 5: Fig. 5** Clinical characteristics analysis of TME-related scores in Caucasian and Asian LUAD patients. (**A-B**) Correlation analysis of TME-related immune score (left), stromal score (middle), estimate score (right), and age in Caucasian (up) and Asian (down) LUAD patients. (**C-D**) Correlation analysis of TME-related immune score (left), stromal score (middle), estimate score (right), and survival status in Caucasian (up) and Asian (down) LUAD patients. (**E–F**) Correlation analysis of TME-related immune score (left), stromal score (middle), estimate score (right), and gender in Caucasian (up) and Asian (down) LUAD patients. (**G-H**) Correlation analysis of TME-related immune score (left), stromal score (middle), estimate score (right), and N stage in Caucasian (up) and Asian (down) LUAD patients. (**I-J**) Correlation analysis of TME-related immune score (left), stromal score (middle), estimate score (right), and smoking status in Caucasian (up) and Asian (down) LUAD patients. (**K-L**) Correlation analysis of TME-related immune score (left), stromal score (middle), estimate score (right), and clinical stage in Caucasian (up) and Asian (down) LUAD patients. (**M–N**) Correlation analysis of TME-related immune score (left), stromal score (middle), estimate score (right), and T stage in Caucasian (up) and Asian (down) LUAD patients.

**Additional file 6: Fig. 6** Differentially expressed genes and functional pathways enrichment analysis related to TME stromal and immune scores in Caucasian and Asian LUAD patients. (**A-B**) Heatmaps of differentially expressed genes related to TME stromal scores in Caucasian (left) and Asian (right) LUAD patients. The abscissa represents patients’ ID, and the ordinate represents gene names. The red bar in the first row represents the group with a lower TME stromal score, and the blue bar represents the group with a higher TME stromal score. The green represents low-expressed genes, and the red represents high-expressed genes. (**C-D**) Heatmaps of differentially expressed genes related to TME immune scores in Caucasian (left) and Asian (right) LUAD patients. The abscissa represents patients’ ID, and the ordinate represents gene names. The red bar in the first row represents the group with a lower TME immune score, and the blue bar represents the group with a higher TME immune score. The green represents low-expressed genes, and the red represents high-expressed genes.

## Data Availability

All the original data in this article are from the online database TCGA (https://www.cancer.gov/) and an article published by Xu JY et al. (https://doi.org/10.1016/j.cell.2020.05.043).
