# Supplementary figures and images for: Comprehensive analysis of PD-L1 expression, tumor-infiltrating lymphocytes, and tumor microenvironment in LUAD: differences between Asians and Caucasians
Source: Clin Epigenetics. 2021 Dec 21;13:229. doi: 10.1186/s13148-021-01221-3 (PMC8693498; doi:10.1186/s13148-021-01221-3)

**A**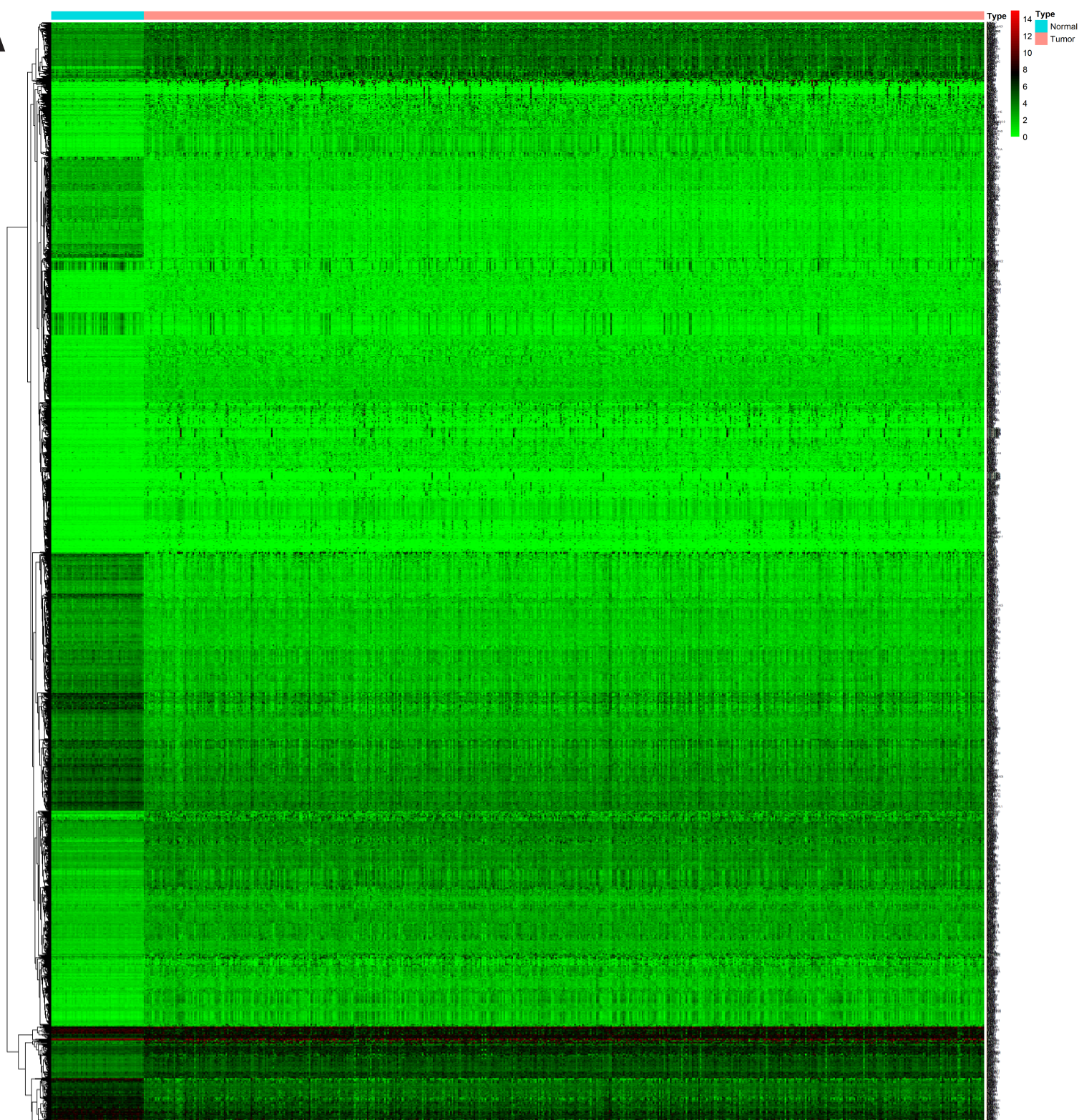**B**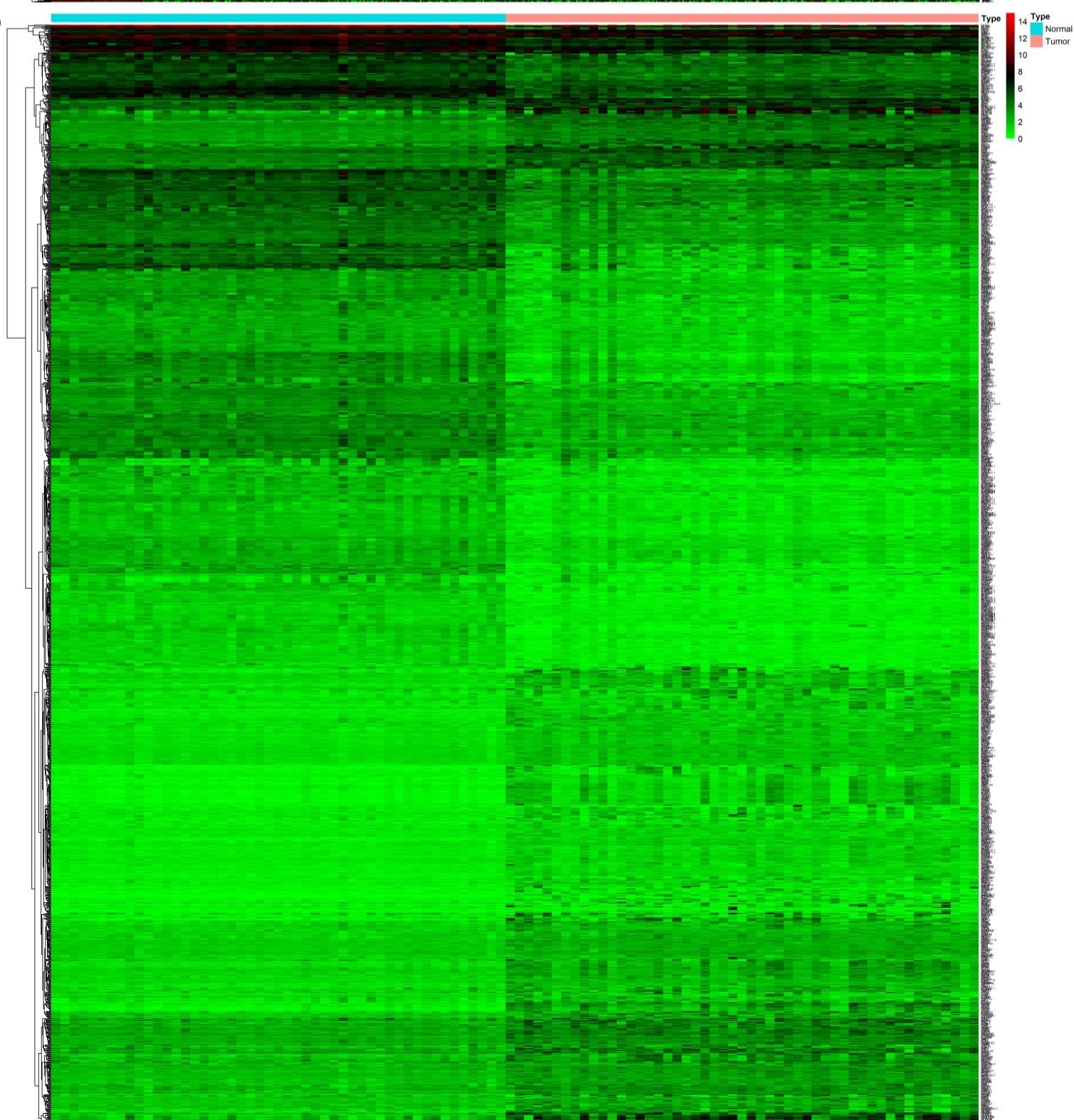

Supplement: Supplementary file 1 — Additional file 1: Fig. 1 Analysis of differentially expressed genes and correlation between PD-L1 expression and prognosis in Caucasian and Asian LUAD patients. (A-B) Differentially expressed gene heatmaps in tumor and normal tissues of LUAD patients in Caucasians (up) and Asians (down). The abscissa represents patients’ ID, and the ordinate represents gene names. The blue bar in the first row represents normal tissue, and the red bar represents tumor tissue. The green represents low-expressed genes, and the red represents high-expressed genes. [file 13148_2021_1221_MOESM1_ESM.pdf]

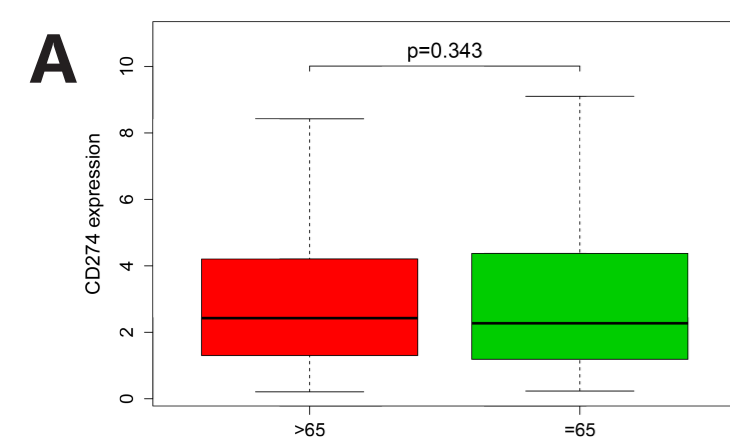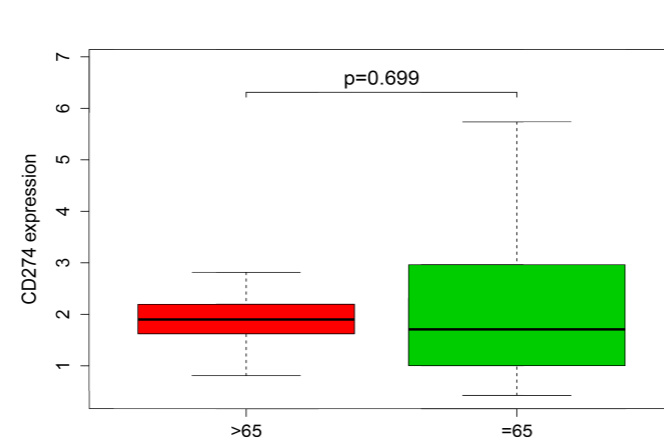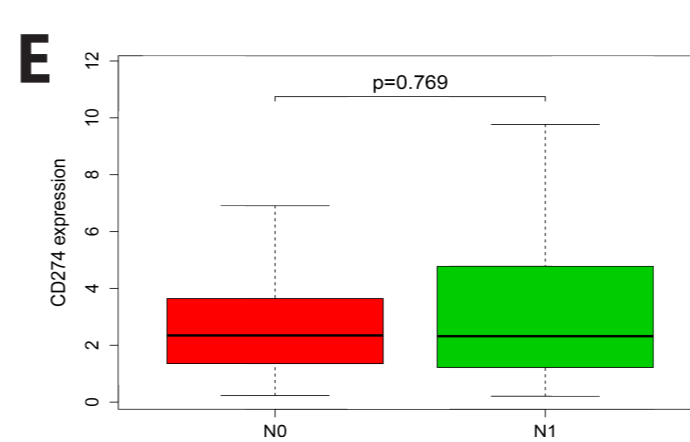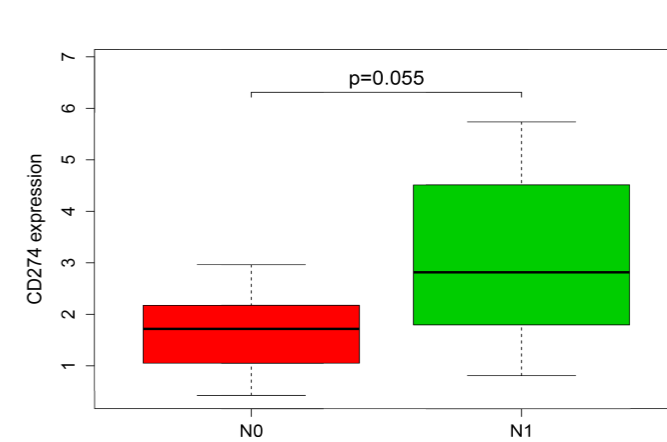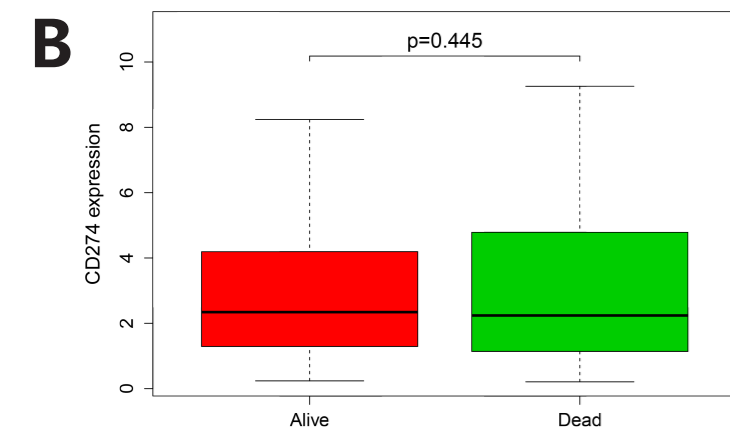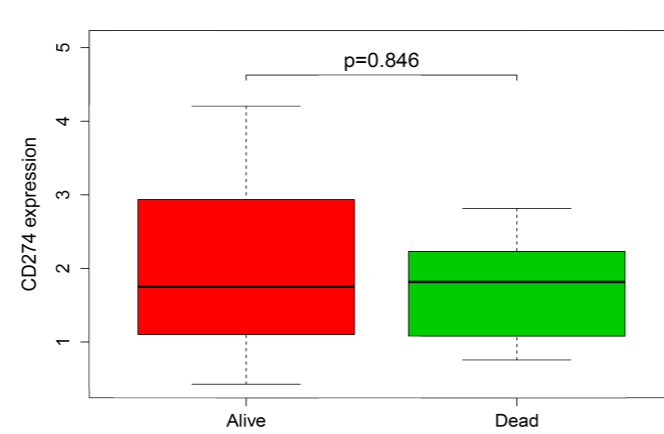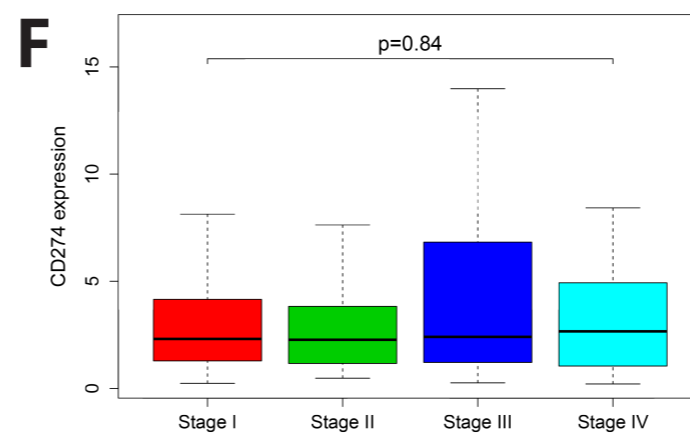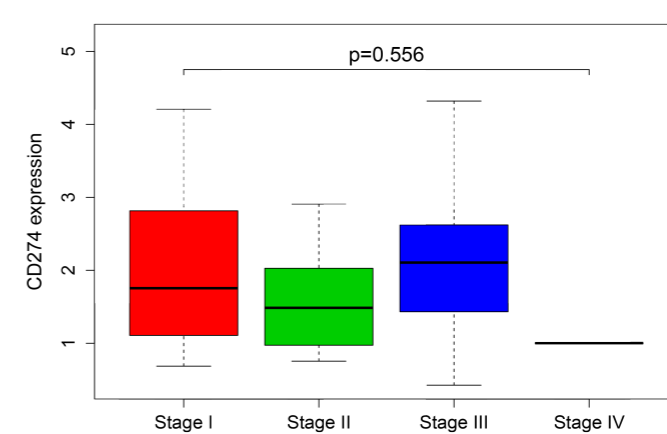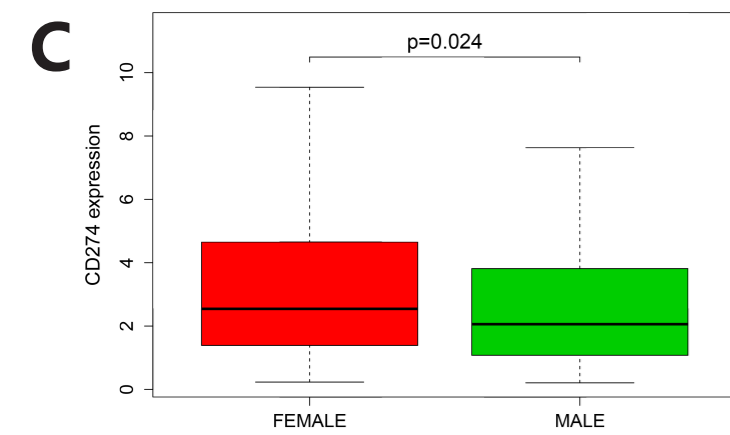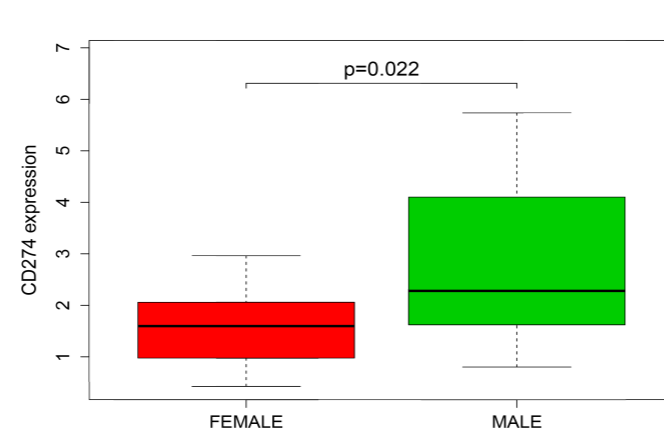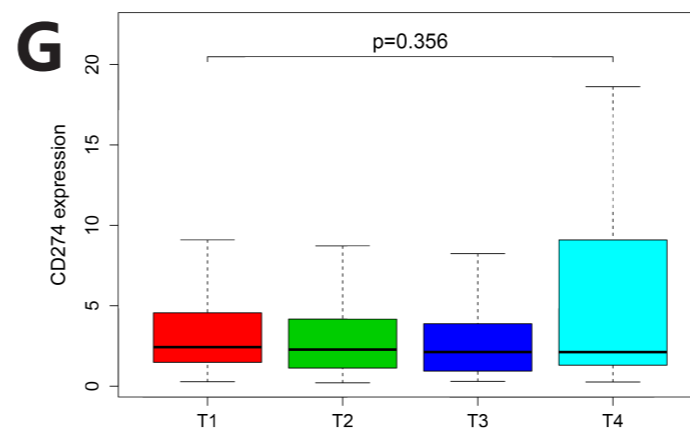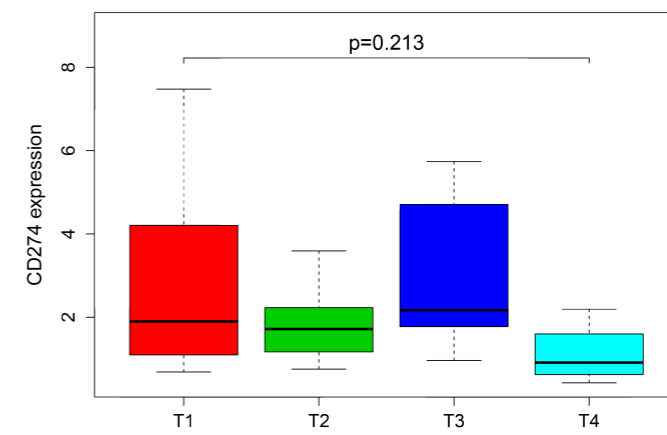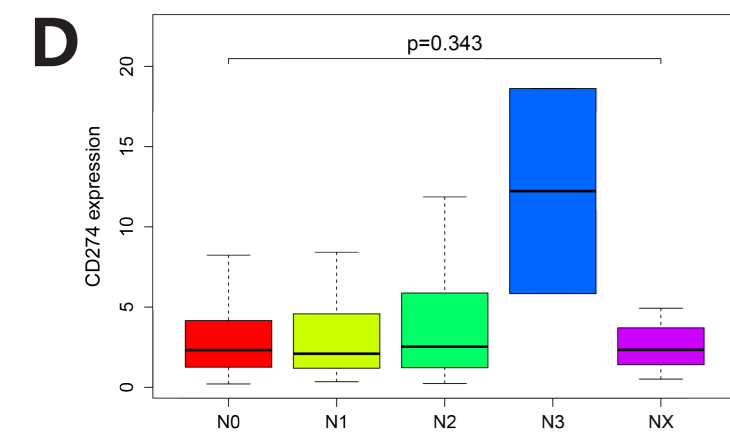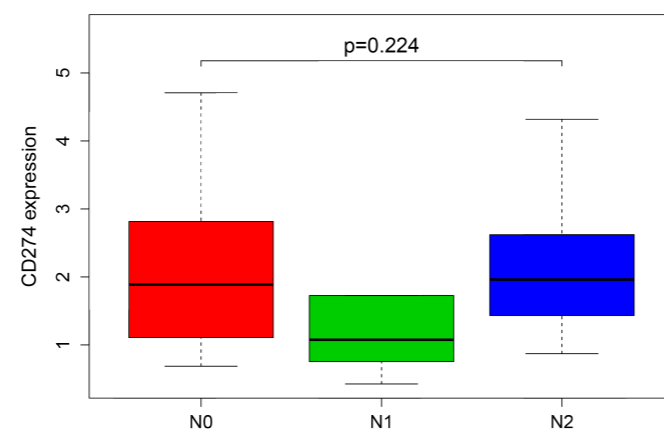

Supplement: Supplementary file 2 — Additional file 2: Fig. 2 Correlation analysis of PD-L1 expression and clinical characteristics in tumor tissues in Caucasian and Asian LUAD patients. (A-G) Barplots of correlation analysis in Caucasian (left) and Asian (right) LUAD patients between PD-L1 expression in tumor tissues and clinical characteristics such as age, survival status, gender, N stage, smoking status, clinical stage, and T stage. The abscissa represents clinical characteristics, and the ordinate represents PD-L1 relative expression. [file 13148_2021_1221_MOESM2_ESM.pdf]

**A**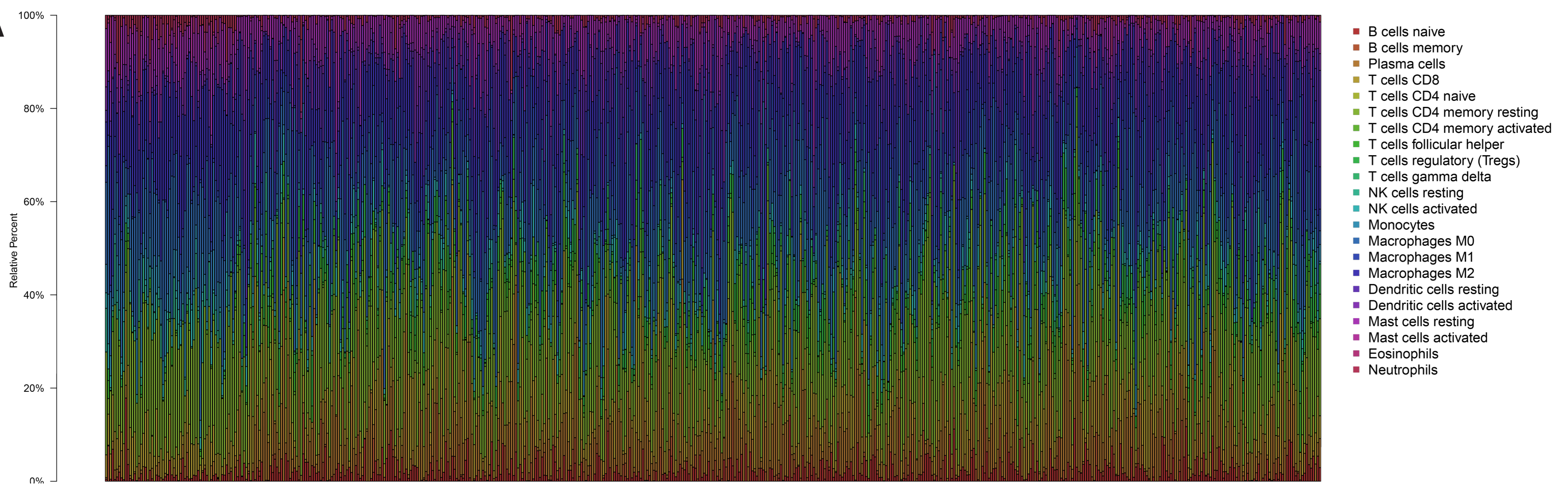**B**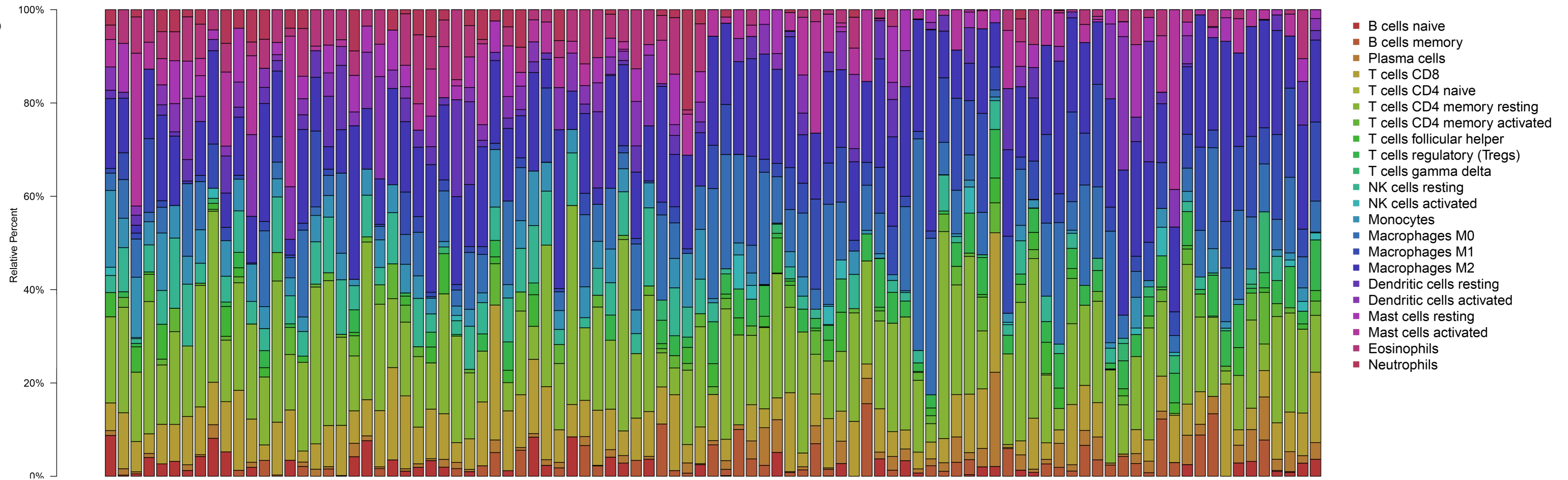

Supplement: Supplementary file 3 — Additional file 3: Fig. 3 Analysis of TILs in Caucasian and Asian LUAD patients. (A-B) Barplots of TILs proportion in Caucasian (up) and Asian (down) LUAD patients. The abscissa represents patients’ ID, and the ordinate represents the percentage of each type of TILs. [file 13148_2021_1221_MOESM3_ESM.pdf]

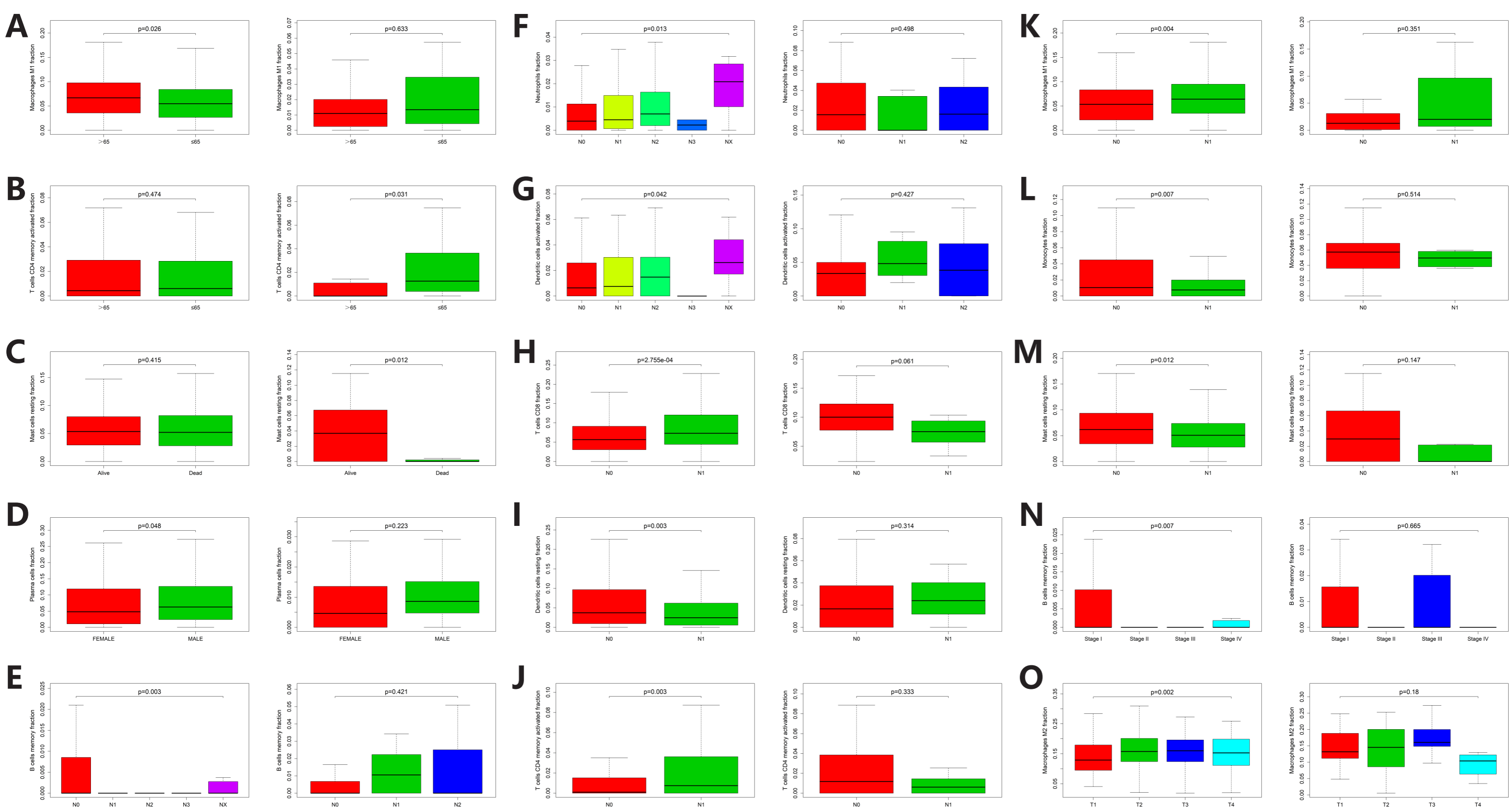

Supplement: Supplementary file 4 — Additional file 4: Fig. 4 Clinical characteristics analysis of TILs in Caucasian and Asian LUAD patients. (A-B) Barplots of correlation analysis between M1 macrophages, activated CD4+ T memory cells, and age in Caucasian (left) and Asian (right) LUAD patients. (C) Barplots of correlation analysis between resting mast cells and survival status in Caucasian (left) and Asian (right) LUAD patients. (D) Barplots of correlation analysis between plasma cells and gender in Caucasian (left) and Asian (right) LUAD patients. (E–G) Barplots of correlation analysis between B memory cells, neutrophils, activated dendritic cells, and N stage in Caucasian (left) and Asian (right) LUAD patients. (H-M) Barplots of correlation analysis between CD8+ T cells, resting dendritic cells, activated CD4+ T memory cells, M1 macrophages, monocytes, resting mast cells, and smoking status in Caucasian (left) and Asian (right) LUAD patients. (N) Barplots of correlation analysis between B memory cells and clinical stage in Caucasian (left) and Asian (right) LUAD patients. (O) Barplots of correlation analysis between M2 macrophages and T stage in Caucasian (left) and Asian (right) LUAD patients. [file 13148_2021_1221_MOESM4_ESM.pdf]

A

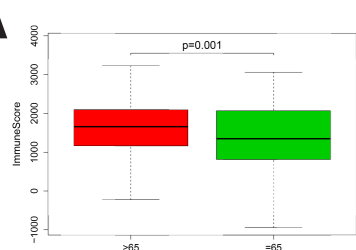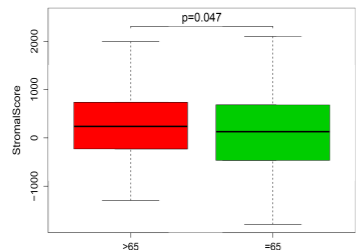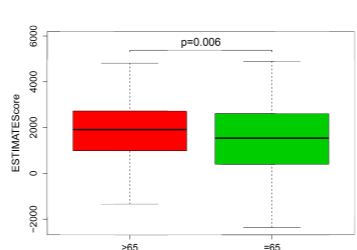

H

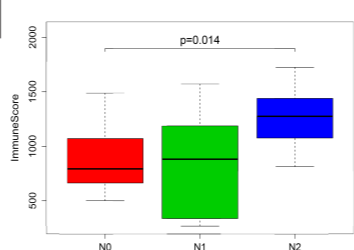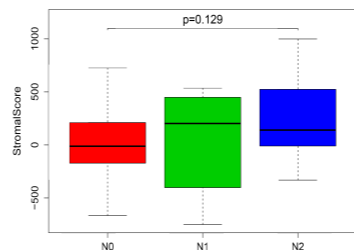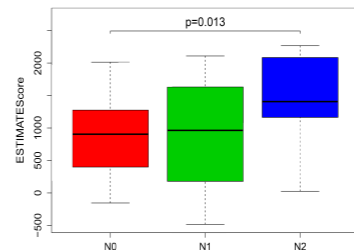

B

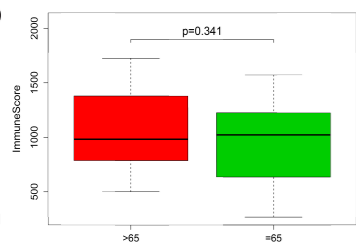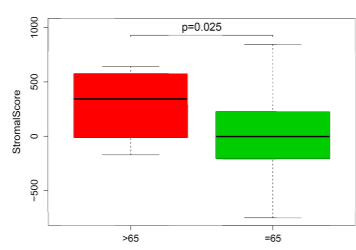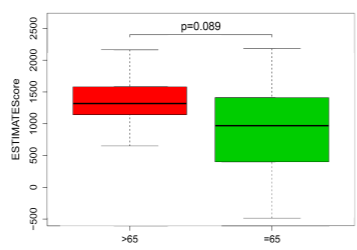

I

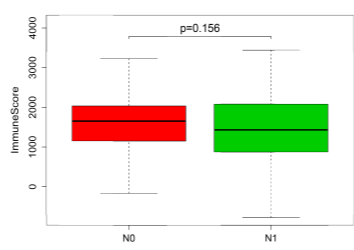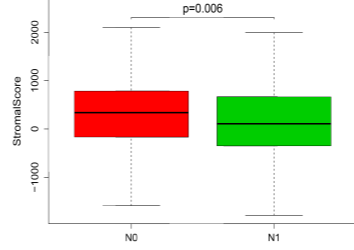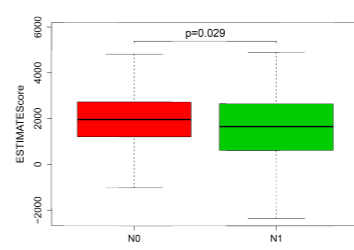

C

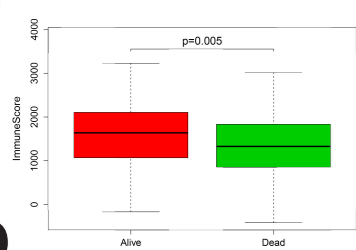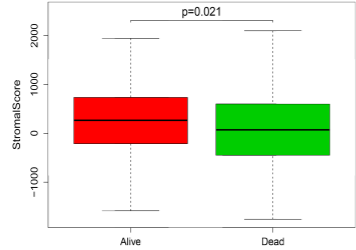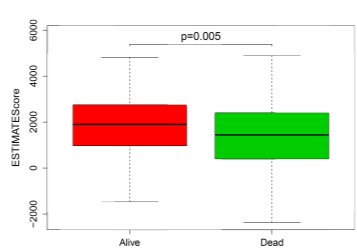

J

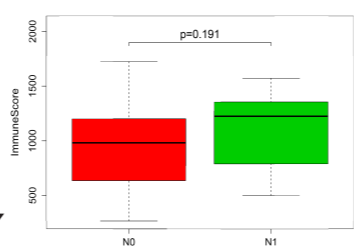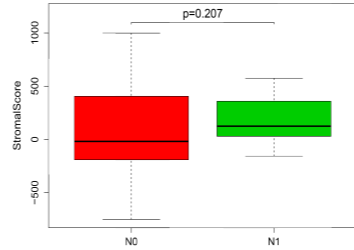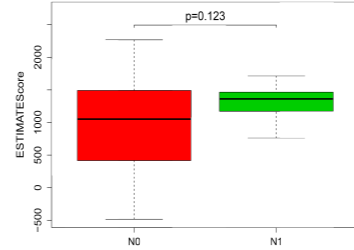

D

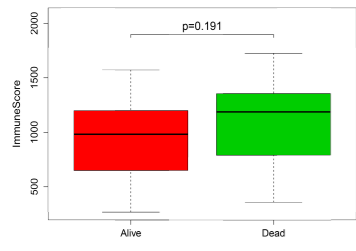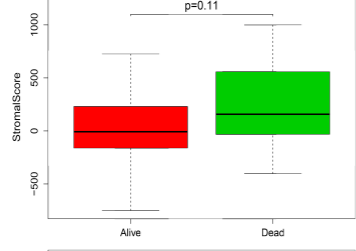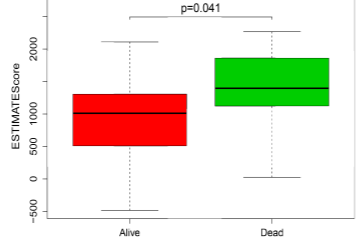

K

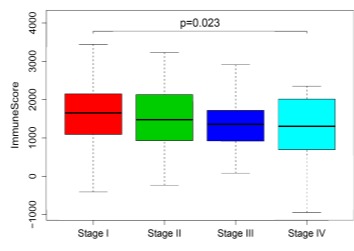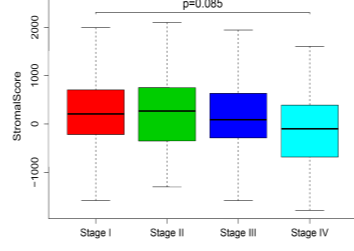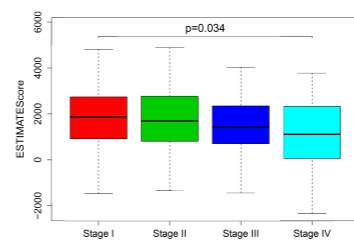

E

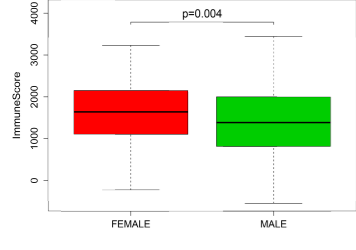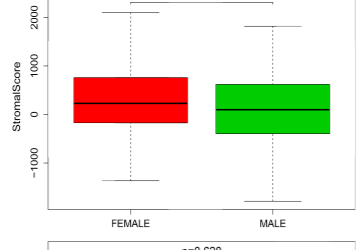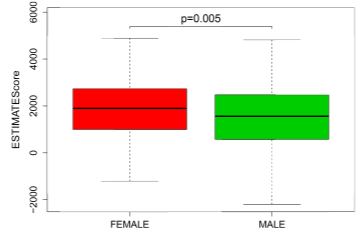

L

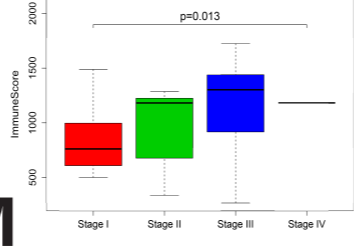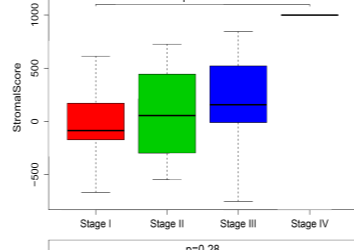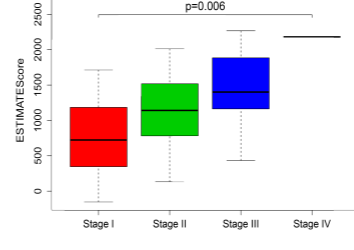

F

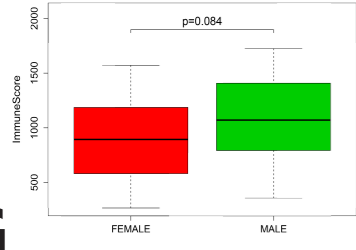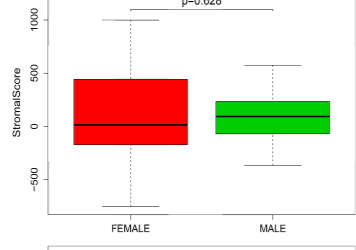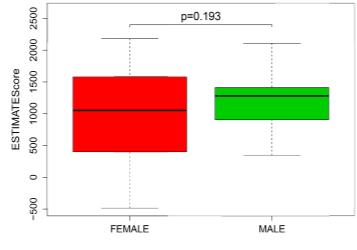

M

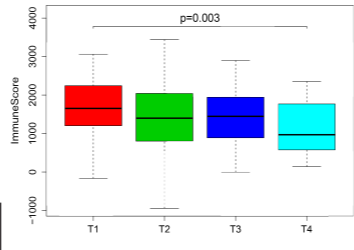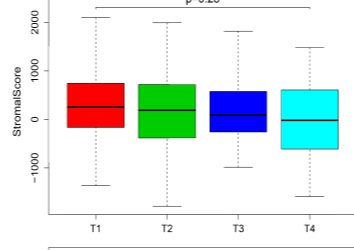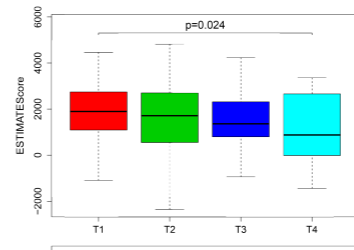

G

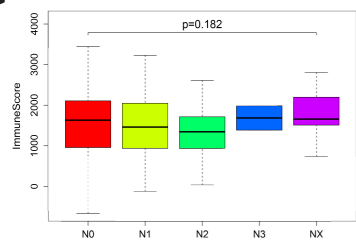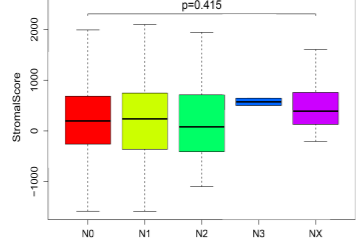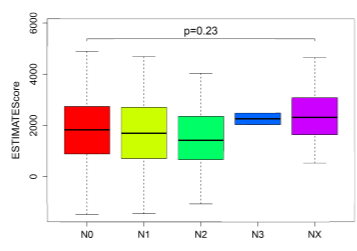

N

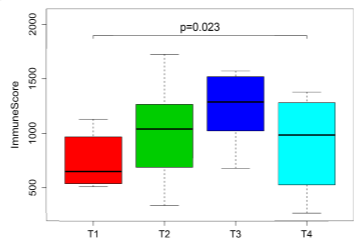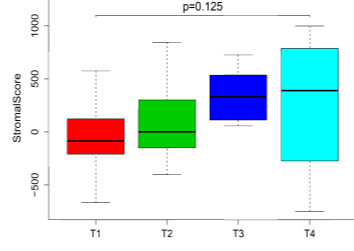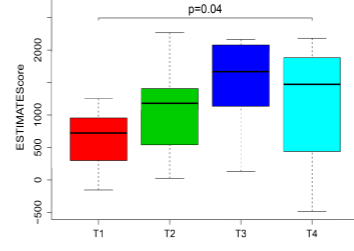

Supplement: Supplementary file 5 — Additional file 5: Fig. 5 Clinical characteristics analysis of TME-related scores in Caucasian and Asian LUAD patients. (A-B) Correlation analysis of TME-related immune score (left), stromal score (middle), estimate score (right), and age in Caucasian (up) and Asian (down) LUAD patients. (C-D) Correlation analysis of TME-related immune score (left), stromal score (middle), estimate score (right), and survival status in Caucasian (up) and Asian (down) LUAD patients. (E–F) Correlation analysis of TME-related immune score (left), stromal score (middle), estimate score (right), and gender in Caucasian (up) and Asian (down) LUAD patients. (G-H) Correlation analysis of TME-related immune score (left), stromal score (middle), estimate score (right), and N stage in Caucasian (up) and Asian (down) LUAD patients. (I-J) Correlation analysis of TME-related immune score (left), stromal score (middle), estimate score (right), and smoking status in Caucasian (up) and Asian (down) LUAD patients. (K-L) Correlation analysis of TME-related immune score (left), stromal score (middle), estimate score (right), and clinical stage in Caucasian (up) and Asian (down) LUAD patients. (M–N) Correlation analysis of TME-related immune score (left), stromal score (middle), estimate score (right), and T stage in Caucasian (up) and Asian (down) LUAD patients. [file 13148_2021_1221_MOESM5_ESM.pdf]

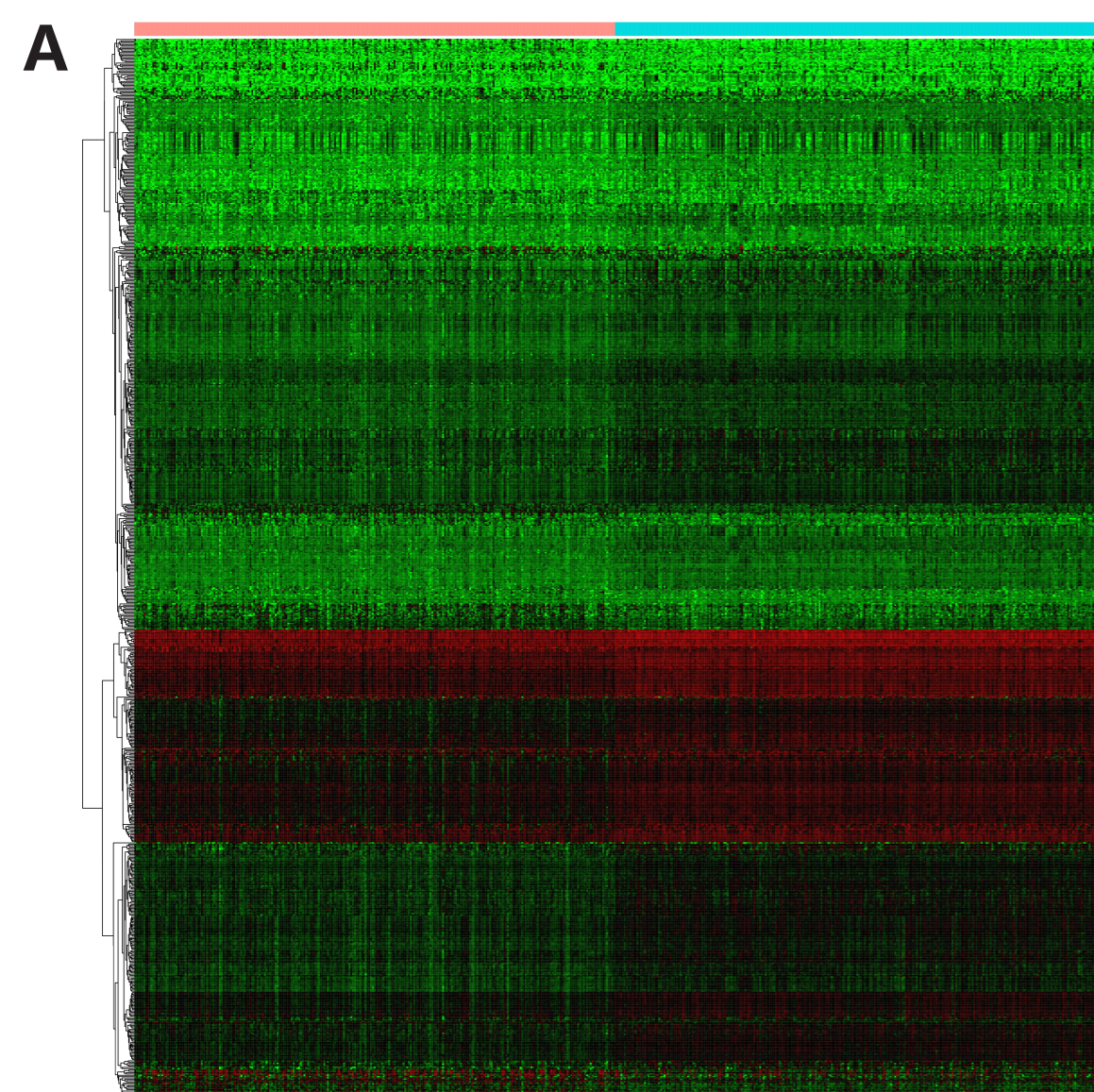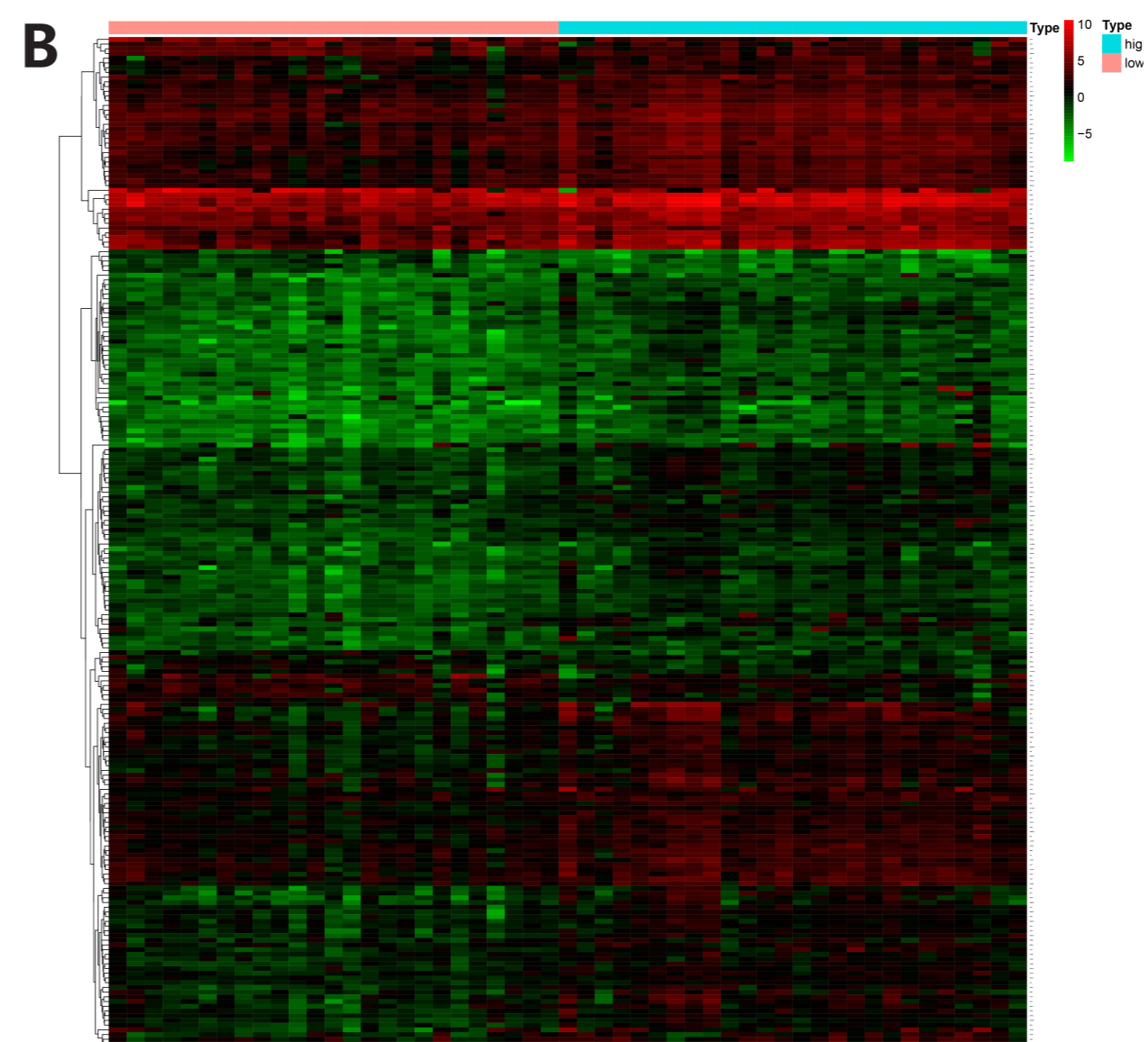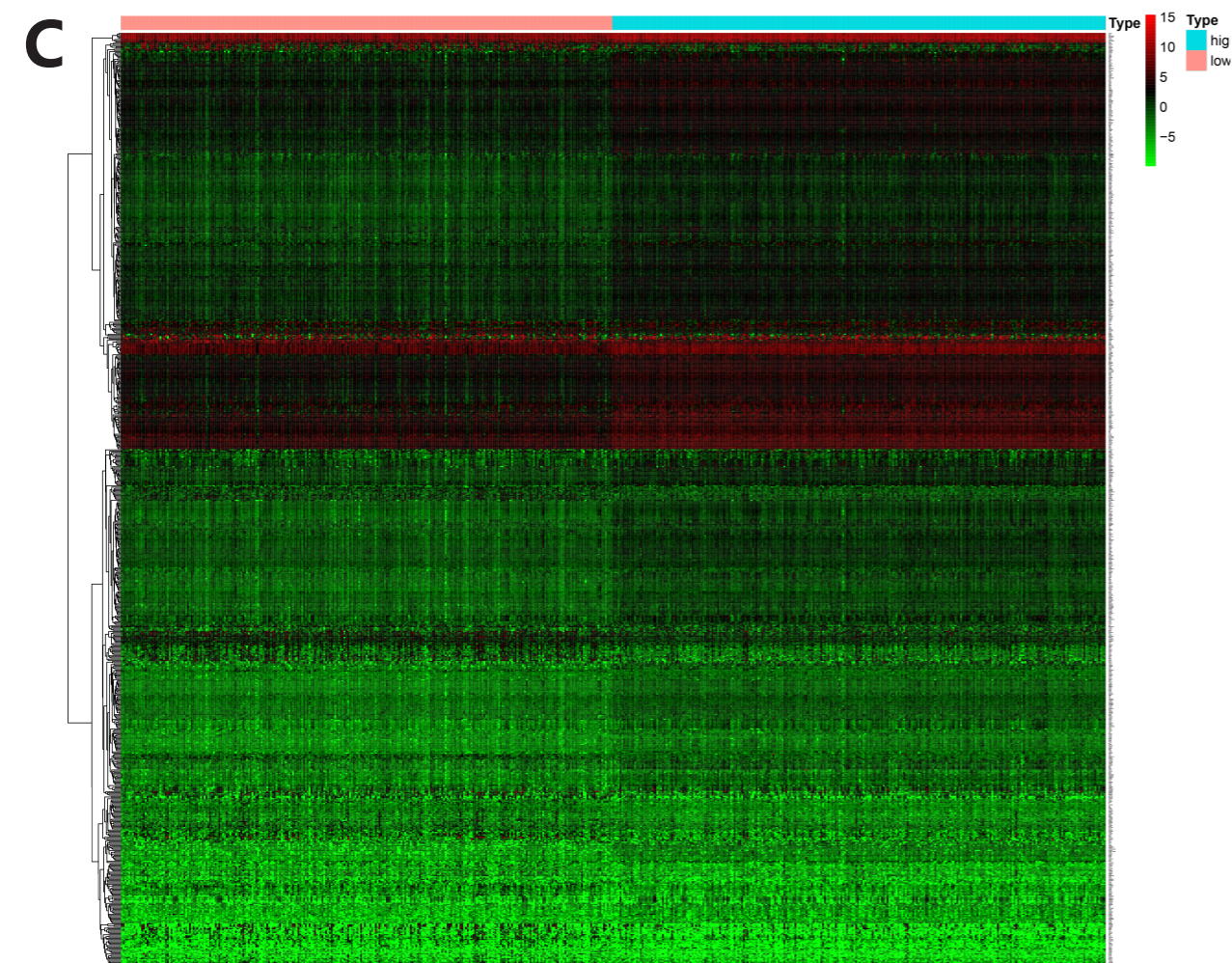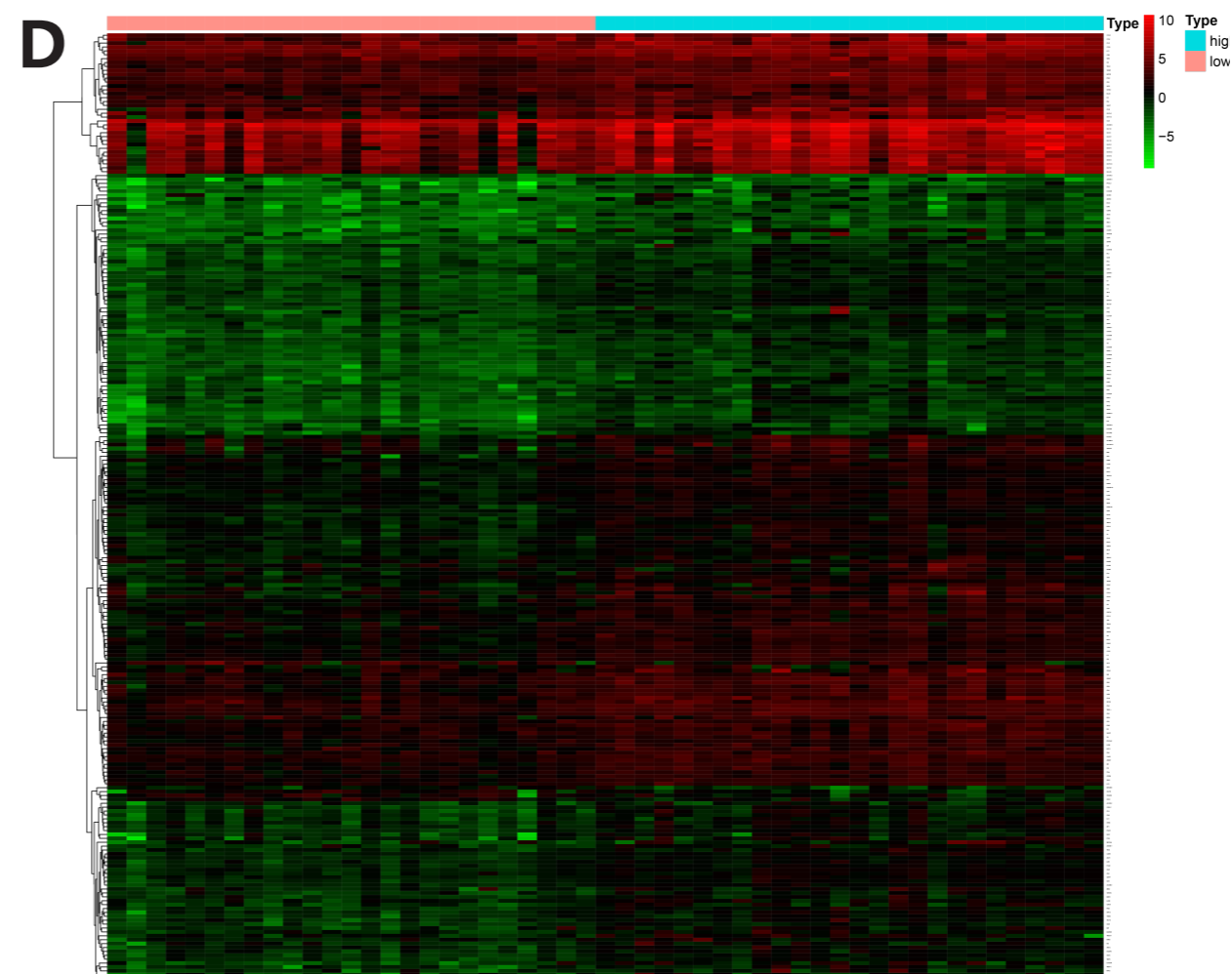

Supplement: Supplementary file 6 — Additional file 6: Fig. 6 Differentially expressed genes and functional pathways enrichment analysis related to TME stromal and immune scores in Caucasian and Asian LUAD patients. (A-B) Heatmaps of differentially expressed genes related to TME stromal scores in Caucasian (left) and Asian (right) LUAD patients. The abscissa represents patients’ ID, and the ordinate represents gene names. The red bar in the first row represents the group with a lower TME stromal score, and the blue bar represents the group with a higher TME stromal score. The green represents low-expressed genes, and the red represents high-expressed genes. (C-D) Heatmaps of differentially expressed genes related to TME immune scores in Caucasian (left) and Asian (right) LUAD patients. The abscissa represents patients’ ID, and the ordinate represents gene names. The red bar in the first row represents the group with a lower TME immune score, and the blue bar represents the group with a higher TME immune score. The green represents low-expressed genes, and the red represents high-expressed genes. [file 13148_2021_1221_MOESM6_ESM.pdf]
